# Murine glomerular transcriptome links endothelial cell-specific molecule-1 deficiency with susceptibility to diabetic nephropathy

**DOI:** 10.1371/journal.pone.0185250

**Published:** 2017-09-21

**Authors:** Xiaoyi Zheng, Fariborz Soroush, Jin Long, Evan T. Hall, Puneeth K. Adishesha, Sanchita Bhattacharya, Mohammad F. Kiani, Vivek Bhalla

**Affiliations:** 1 Division of Nephrology, Department of Medicine, Stanford University School of Medicine, Stanford, California, United States of America; 2 Department of Mechanical Engineering, College of Engineering, Temple University, Philadelphia, Pennsylvania, United States of America; 3 Institute of Computational Health Sciences, University of California, San Francisco, California, United States of America; Baker IDI Heart and Diabetes Institute, AUSTRALIA

## Abstract

Diabetic nephropathy (DN) is the leading cause of kidney disease; however, there are no early biomarkers and no cure. Thus, there is a large unmet need to predict which individuals will develop nephropathy and to understand the molecular mechanisms that govern this susceptibility. We compared the glomerular transcriptome from mice with distinct susceptibilities to DN at four weeks after induction of diabetes, but before histologic injury, and identified differential regulation of genes that modulate inflammation. From these genes, we identified endothelial cell specific molecule-1 (Esm-1), as a glomerular-enriched determinant of resistance to DN. Glomerular Esm-1 mRNA and protein were lower in DN-susceptible, DBA/2, compared to DN-resistant, C57BL/6, mice. We demonstrated higher Esm-1 secretion from primary glomerular cultures of diabetic mice, and high glucose was sufficient to increase Esm-1 mRNA and protein secretion in both strains of mice. However, induction was significantly attenuated in DN-susceptible mice. Urine Esm-1 was also significantly higher only in DN-resistant mice. Moreover, using intravital microscopy and a biomimetic microfluidic assay, we showed that Esm-1 inhibited rolling and transmigration in a dose-dependent manner. For the first time we have uncovered glomerular-derived Esm-1 as a potential non-invasive biomarker of DN. Esm-1 inversely correlates with disease susceptibility and inhibits leukocyte infiltration, a critical factor in protecting the kidney from DN.

## Introduction

Diabetic nephropathy (DN) is the most common cause of chronic kidney disease and end-stage renal disease in the developed and developing world[1]. Despite clinical trials affirming the importance of glycemic control (in type 1 diabetes) and the inhibition of the renin-angiotensin-aldosterone system (in both type 1 and type 2 diabetes) for slowing progression of nephropathy, beneficial effects are modest[2], and the overall burden of DN continues to increase[3]. Moreover, an increased prevalence of type 2 diabetes[2] and the high cardiovascular risk associated with kidney disease[4] suggest that DN will absorb a disproportionate fraction of health care resources in the coming decades.

Among diabetic complications, DN is associated with the highest cardiovascular morbidity and mortality[5]. However, in patients with either type 1 or type 2 diabetes, the prevalence of nephropathy, defined as macroalbuminuria with or without reduced glomerular filtration rate is less than 15% [6, 7]. Diabetic nephropathy is characterized by changes in the glomerulus including mesangial matrix accumulation[8], podocyte apoptosis[9], endothelial cell injury[10], and leukocyte infiltration[11], all of which can contribute to albuminuria and/or impaired function[8, 11, 12]. While genes responsible for initiation and/or progression of DN have not been definitively identified, genetic predisposition to the disease is thought to play a major role[13, 14].

The NIH-sponsored Diabetic Complications Consortium has phenotypically characterized the clinical response to hyperglycemia among genetically distinct inbred mouse strain[15, 16]. One strain (DBA/2, DN-susceptible) develops albuminuria compared with another more widely used strain (C57BL/6, DN-resistant). Several investigators have shown that DBA/2 mice treated with the β-islet cell toxin, streptozotocin, exhibit increased mesangial matrix accumulation, podocyte apoptosis, and leukocyte infiltration compared with C57BL/6 mice that have similar levels of hyperglycemia[15–17]. The DBA/2 background is also permissive for DN in genetic models of diabetes, Akita and *db/db* mice[18, 19].

We hypothesized that by comparing glomerular transcripts from these differentially susceptible mice, we would identify genes that regulate susceptibility to DN. We sought to study initiating factors of disease in the glomerular transcriptome. Thus, we analyzed genes from these two strains that were differentially regulated by diabetes, four weeks after the onset of diabetes but before the onset of kidney injury [20]. From these transcripts, validation of gene expression and function would inform molecular mechanisms and potential new therapeutic targets for DN.

## Materials and methods

### Animals

We purchased seven week-old DBA/2 and C57BL/6 male mice from Jackson Laboratory and housed these mice in the Stanford University Veterinary Service Center. We then induced diabetes at 8 weeks of age per the Diabetic Complications Consortium protocol [16].with low dose streptozotocin (Sigma Aldrich, St. Louis, MO, USA) for five consecutive days. After four or six weeks, we measured six hour fasting tail vein blood glucose to validate hyperglycemia (Contour, Whippany, NY, USA). We collected overnight mouse urine at the times indicated, and measured urine albumin by ELISA (Exocell, Philadelphia, PA, USA) and urine creatinine by HPLC/MS/MS (Mouse Metabolic Phenotyping Center, Yale University, New Haven, CT, USA). We collected sera by retro orbital bleeding (Kimble Chase, Vineland, NY, USA) immediately before sacrifice.

### Isolation and culture of glomeruli from mouse kidney

We inactivated Dynabeads M-450 (Invitrogen, Carlsbad, CA, USA) with 1 mL 0.1% BSA/0.2M Tris (pH 8.5) at 37°C overnight and then perfused each mouse with 8 x 10^7^ beads in 35–40 mL PBS. We dissociated kidney cells by 1mg/mL collagenase A in 1 mL Dulbecco`s PBS, 37°C for 30 minutes and passed the digested material through a 100 μM cell strainer (Fischer Scientific, Waltham, MA, USA) and isolated glomeruli by a DynaMag-2 magnetic particle concentrator (Invitrogen, Carlsbad, CA, USA). The specificity of isolated glomerular tissue is shown in S1 Fig. We dissolved glomeruli in Tri-reagent (Sigma Aldrich, St. Louis, MO, USA) for RNA preparation, or plated in 1 mL of DMEM (Mediatech, Tewksbury, MA, USA) supplemented with 0.2% FCS (Fischer Scientific, Waltham, MA, USA) in 24-well tissue culture plates and incubated for 24 hours in low (100 mg/dL) or high glucose media (450 mg/dL). We then collected the conditioned media for analysis of Esm-1.

For experiments to separate glomerular and tubulointerstitial fractions, we averted the need for Dynabeads by digesting kidney in 2mg/mL collagenase A in Hepes Ringer Buffer, 37°C for 120 minutes. We then filtered the digested material through a 40 μM cell strainer to collect tubulointerstitial fragments and then filtered the glomeruli on a 100μM cell strainer. We dissolved the glomeruli and tubulointerstitial fractions in Tri-reagent for RNA preparation. The specificity of isolated kidney compartments is shown in S2 Fig.

### Microarray analysis

We applied 1 μg of glomerular RNA to an Illumina array (Affymetrix, Santa Clara, CA, USA) to analyze differentially transcribed genes. We compared the mean signal intensity from glomerular RNA of control or diabetic DBA/2 or C57BL/6 mice (N = 4 mice per group) with the referent group as indicated. We submitted the microarray data set to the Gene Expression Omnibus, with accession number GSE84663 (http://www.ncbi.nlm.nih.gov/geo/).

### Gene enrichment analysis

We identified sets of significantly differentially expressed genes, which were mapped to the GeneGo database by MetaCore (lsresearch.thomsonreuters.com). In MetaCore, we calculated the p-value using a hypergeometric distribution. We defined the number of intersecting objects in the experiment as r, the number of network objects in the experiment as n, the total number of intersecting network objects in the database as R, and the total number of network objects in the database as N. We calculated a p-value and false discovery rate for each object in the experiment based on its number of intersections.

### Tissue expression analysis of human Esm-1

We obtained publicly available RNAseq data maintained by the European Bioinformatics Institute (http://www.ebi.ac.uk/)[21]. From seven existing datasets, we pooled raw expression counts (Fragments Per Kilobase of transcript per Million mapped reads), pooled similar organs that varied anatomically (e.g. left kidney and right kidney; arm muscle and leg muscle) as duplicates, and compared the median expression across different tissues.

### Quantitative PCR

We used ImpromII Reverse Transcriptase (Promega, Madison, WI, USA) to prepare cDNA from total tissue RNA per the manufacturer’s instructions. We then amplified cDNA by using the qPCR master mix (Applied Biosystems, Grand Island, NY, USA) and the StepOne Plus Real Time qPCR system (Applied Biosystems, Grand Island, NY, USA) with the following protocol: heat activation: 95°C 20s, denaturation 95°C 3s, extension: 60°C 30s, 40 cycles. We used the following primers: Cdh5, forward: TGGTCACCATCAACGTCCTA, reverse: ATTCGGAAGAATTGGCCTCT. CD31, forward: GACCCAGCAACATTCACAGATA, reverse: ACAGAGCACCGAAGTACCATTT. Esm-1, forward: GGCGATAAAACAAGACCAGAAA, reverse: AAACCAGAGATGAGAAGTGATGG. Midkine, forward: AGCCGACTGCAAATACAAGTTT, reverse: GCTTTGGTCTTTGACTTGGTCT. Nephrin, forward: TGCTGCCTTACCAAGTCCAG, reverse: GCTTCTGGGCCGGGTATTTT. SGLT2, forward: TGGCGGTGTCCGTGGCTTGG, reverse: CGGACACTGGAGGTGCCAGATAGC. Tsc22d3, forward: AAGCAACCTCTCTCTTCTTCTCTG, reverse: ATAAGCAGTCATCCCAAAGCTGTA.

### Esm-1 ELISA

The level of mouse serum Esm-1 is approximately 1.0 ng/mL[22]. Therefore, we analyzed mouse serum samples using a Mouse Esm-1 ELISA Kit with a detection range of 23-1500pg/mL (Aviscera Biosciences, Santa Clara, CA, USA). We normalized the urine Esm-1 concentration to creatinine, a quantitative control for glomerular filtration rate. Positive and negative controls for the Esm-1 ELISA are provided in S3 Fig.

### Biomimetic microfluidic assay (bMFA)

#### Plating endothelial cells in the microfluidic chip

With the use of our established protocol[23], we coated the chip with fibronectin (100 μg/mL) for 60 minutes and plated human umbilical vein endothelial cells (HUVEC) (Lonza, Walkersville, MD, USA) into the bMFA. After 4 to 6 hours, we applied shear flow to form a three dimensional lumen in the vascular channels. We next activated confluent endothelial cells with 10 units/mL of TNF-α for 4 hours.

#### Leukocyte isolation and labeling

We obtained human blood from healthy adult donors in sodium heparin (BD Biosciences, San Jose, CA, USA), and isolated neutrophils by a one-step Ficoll-Plaque gradient (GE Healthcare, Piscataway, NJ, USA). We next resuspended neutrophils in Hanks Balanced Salt Solution (HBSS) (5 × 10^6^ cells/mL) and labeled with Carboxyfluorescein Diacetate, Succinimidyl Ester probe (Molecular Probes, Carlsbad, CA, USA) for 10 minutes at room temperature. After activation by 10 units/mL of TNF-α for 10 minutes, we incubated neutrophils were with media or recombinant human Esm-1 (R&D systems, Minneapolis, MN, USA) for 10 minutes at room temperature.

#### Leukocyte-endothelial interaction under shear flow

We filled the tissue compartment of the bMFA with chemotactic, N-Formylmethionine-leucyl-phenylalanine (1 μM; Sigma Aldrich) prior to the experiments. The fixed flow rate at 1 μL/min injects 5000 Carboxyfluorescein Diacetate, Succinimidyl Ester probe labeled neutrophils per minute, at 37°C. With a previously developed computational fluid dynamics (CFD)-based model [23], we calculated shear rates in different channels of the network. We recorded video clips at 30 fps using a Rolera Bolt camera (QImaging, Surrey, BC, Canada). After 10 minutes of flowing neutrophils into the bMFA, we injected PBS from the inlet port for 5 minutes to completely wash off unbound neutrophils. We scanned the entire bMFA at the 10× objective using an automated stage on an epifluorescence microscope (Nikon Eclipse TE200, Melville, NY, USA). We processed the acquired images and videos using Nikon Elements software.

#### Data analysis

We quantified the numbers of rolling, adherent, and migrating leukocytes in the bMFA using Nikon Elements software. We considered any leukocyte traveling at a velocity below the critical velocity as a rolling cell. We estimated critical velocity from a cell velocity flowing in the centerline (v_cc_) as v_crit_ = v_cc_ × ε × (2 − ε), where ε is the cell-to-vessel diameter ratio. We considered any cell that did not move for 30 seconds as adherent.

### Statistics

For analysis of the microarray data, we normalized expression patterns using quantile normalization in R statistical software and deemed differences as significant if there were ≥ 2-fold change between groups if the p-value (adjusted for multiple comparisons using the Benjamini–Hochberg procedure) was below 0.05.

For analysis of human tissue RNAseq data, due to the skewness and heteroscedasity of the raw counts data, we performed the Mann-Whitney test to compare Esm-1, CD31, and Cadherin 5 expression levels in kidney with the other five highest Esm-1-expressing tissues (lung, thyroid, aorta, adrenal gland, and tibial artery). We deemed differences to be statistically significant if the p-value (adjusted for multiple comparisons using a Bonferroni correction) was below 0.05.

For all other experiments, we used Student’s t-test to compare two experimental groups, and one- or two-way analysis of variance to compare three or more experimental groups. We expressed the results as mean +/- standard error of the mean and deemed differences to be statistically significant if the p-value was below 0.05.

### Study approval

The Institutional Animal Care and Use Committee of the Stanford University School of Medicine approved all experiments. For human samples, written informed consent was obtained as approved by the Institutional Review Board of Temple University.

## Results

### Glomerular expression patterns significantly differ between DN-susceptible and DN-resistant mice

To better understand the susceptibility to kidney disease, we compared glomerular gene expression profiles from DN-susceptible and DN-resistant mice (DBA/2 vs. C57BL/6 mice, respectively) four weeks after induction of diabetes by low-dose streptozotocin[15, 16] vs. vehicle-treated non-diabetic controls (S4 Fig). Prior studies have elucidated these strains as DN-susceptible or DN-resistant[15, 16] by histology and albuminuria, and we reaffirmed this classification by albuminuria at 16 weeks after induction, in separate cohorts of mice (S5 Fig).

**Fig 1:**
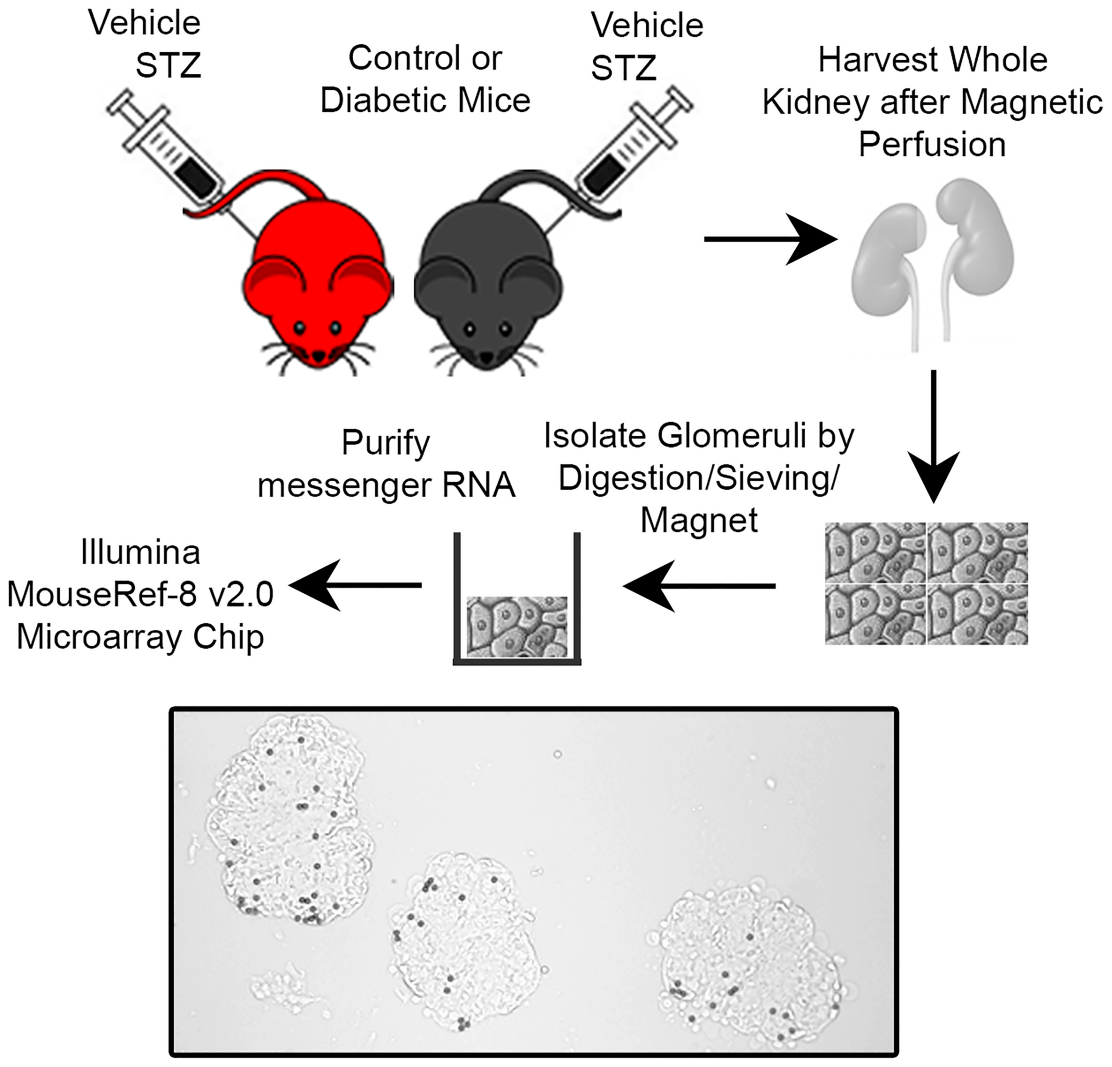
Protocol for comparison of differentially expressed glomerular RNA between DN-susceptible and DN-resistant mice. DN-susceptible (*red*) and DN-resistant (*black*) mice were injected with vehicle or streptozotocin (STZ), and after four weeks, glomerular RNA was isolated for microarray analysis. A representative brightfield image of isolated glomeruli after magnetic bead perfusion is shown, 200x magnification. N = 4 mice per group.

**Fig 2:**
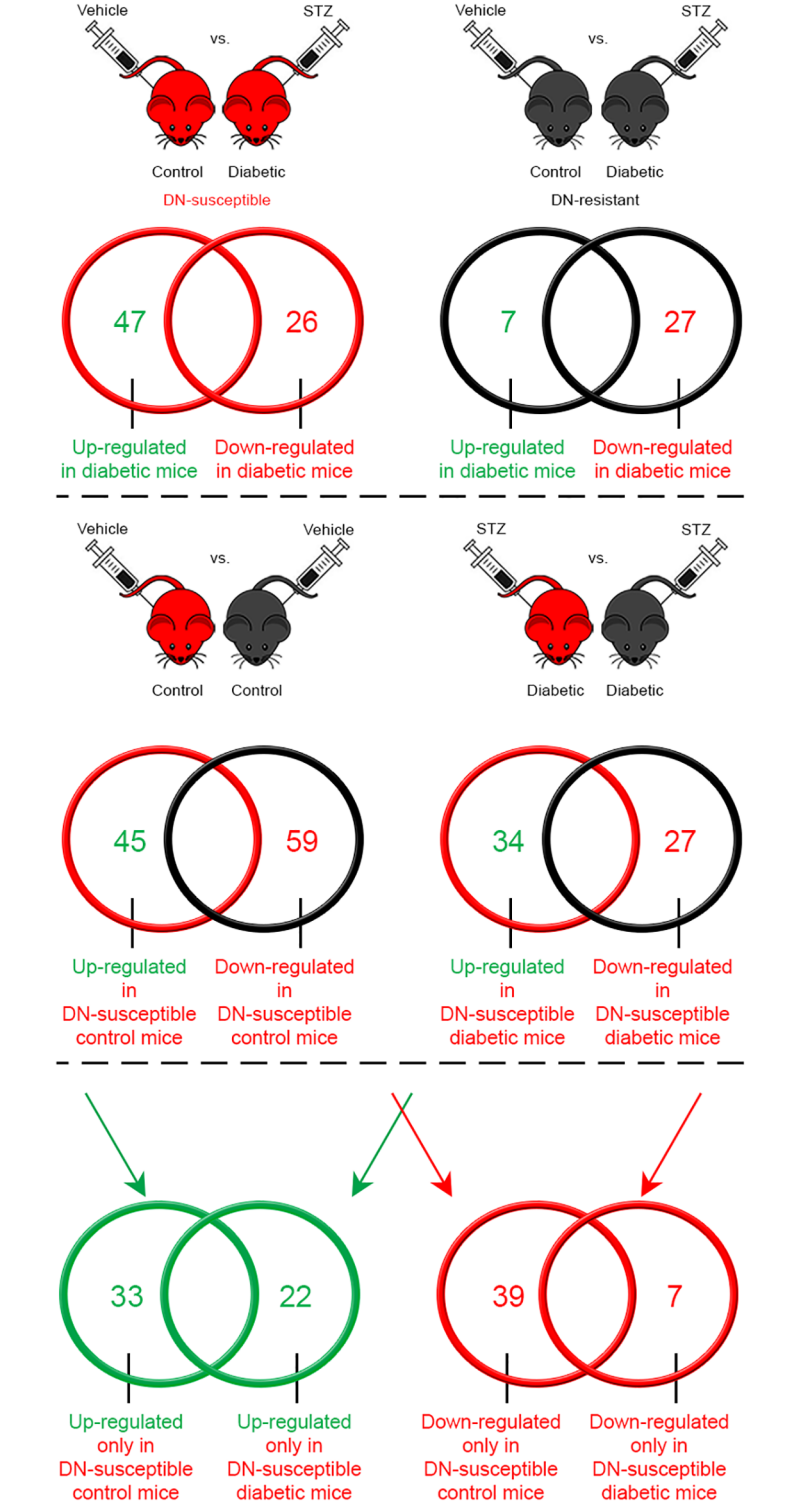
Comparison of differentially expressed glomerular RNA between control and diabetic DN-susceptible and DN-resistant mice. Venn diagrams showing the number of significantly up- and down-regulated genes in each comparison relative to the indicated referent group. Diabetic vs. control (referent group) DN-susceptible mice, *upper left panel*; Diabetic vs. control (referent group) DN-resistant mice, *upper right panel*; control DN-susceptible vs. DN-resistant mice (referent group), *middle left panel*; diabetic DN-susceptible vs. DN-resistant mice (referent group), *middle right panel*. The number of up- (*lower left panel*) and down-regulated (*lower right panel*) genes exclusively in control or diabetic DN-susceptible vs. DN-resistant (referent group) mice. The number of up- and down-regulated genes are shown in *green* and *red*, respectively. The diagrams from DN-susceptible vs. DN-resistant mice are shown in *red* vs. *black*, respectively. N = 4 mice per group.

After extraction of total glomerular RNA and analysis by microarray (Fig 1), we first compared gene expression in two ways (Fig 2, ***top panel***): 1) control vs. diabetic DN-susceptible mice and 2) control vs. diabetic DN-resistant mice. We restricted our analysis to unique gene identifiers in which the mean fold change was greater or less than 2, and the genes were significantly different between groups. There were 47 genes up-regulated and 26 genes down-regulated by diabetes in DN-susceptible mice (S1 Table). In DN-resistant mice, there were only 7 genes up-regulated and 27 genes down-regulated by diabetes (S3 Table). We also performed pathway analysis for these gene sets (S2 and S4 Tables, respectively) and show that immune system pathways were consistently and significantly highlighted in diabetic vs. control mice in both strains, indicating that the immune system is a feature influenced by diabetes.

In order to study strain-specific differences in susceptibility and to focus on genes that were differentially regulated by diabetes within the two strains, we next compared 1) control DN-susceptible vs. DN-resistant mice and 2) diabetic DN-susceptible vs. DN-resistant mice (Fig 2, ***middle panel***). We again restricted our analysis to unique gene identifiers in which the mean fold change was greater or less than 2, and the genes were significantly different between groups. There were 45 genes up-regulated and 59 genes down-regulated in control DN-susceptible mice, relative to DN-resistant mice (S5 Table). Among diabetic mice, there were 34 genes up-regulated and 27 genes down-regulated by diabetes (S7 Table). We also performed pathway analysis for differentially-expressed genes in control DN-susceptible vs. DN-resistant mice (S6 Table). Several non-immune system pathways including protein folding and apoptosis highlight the primary background differences between these mouse strains. In contrast, in diabetic DN-susceptible vs. DN-resistant mice (S8 Table), immune system pathways were among the most significantly different transcriptional programmes.

**Fig 3:**
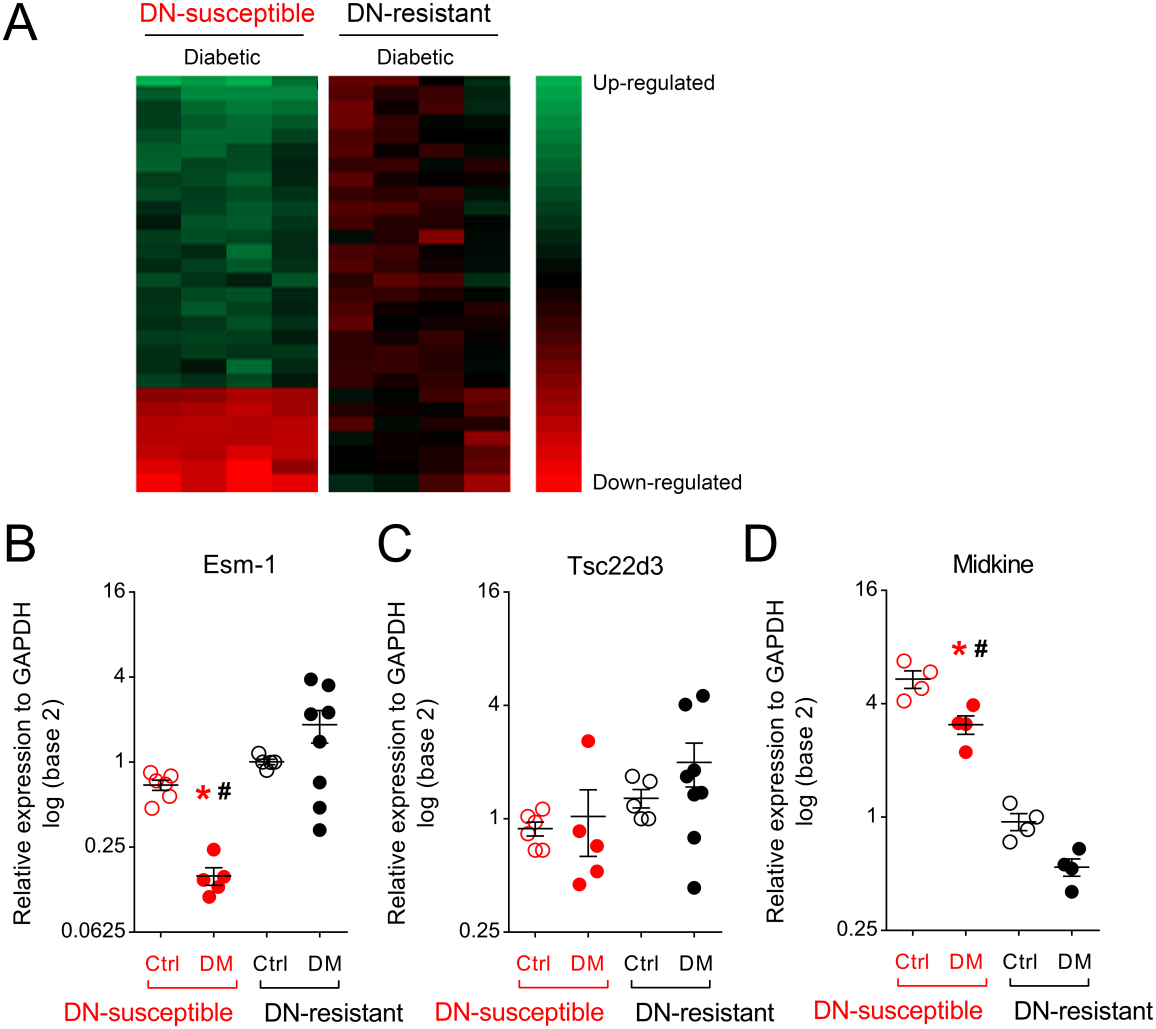
Heat map and validation of differential expression of 3 immune-related genes from microarray results. (A) A microarray analysis (heat map) aligned by significantly different, unique gene identifiers for which the mean was > or < 2 –fold more abundant in glomerular RNA from diabetic DN-susceptible vs. DN-resistant mice. Each column represents the values of each sample relative to the mean expression level from diabetic DN-resistant mice. Each row represents a separate gene from the microarray. Genes with the higher and lower expression in DN-susceptible vs. DN-resistant samples are shown in *green* and *red*, respectively. Genes colored in *black* were no different from the mean of the control group. Quantitative PCR of relative gene expression from DN-susceptible and DN-resistant mice for: (B) Esm-1, (C) Tsc22d3, and (D) Midkine. Samples from control, DN-resistant mice are used as the reference group. Each circle represents data from one mouse. Open and closed circles indicate data from vehicle- and streptozotocin-injected (i.e. control and diabetic) mice, respectively. Red and black circles/asterisks indicate data from DN-susceptible and DN-resistant mice, respectively. Ctrl, control; DM, diabetic. *, p-value < 0.05 in the same mouse strain between control and diabetic groups. #, p-value < 0.05 in the same treatment between the two mouse strains. N = 4–8 mice per group; n = 3 replicate wells per sample.

To further refine the analysis, we examined only genes that were significantly different between diabetic DN-susceptible vs. DN-resistant mice, but not between control groups (Fig 2, ***bottom panel***). We therefore identified 22 unique significantly higher-expressed genes and 7 lower-expressed genes between diabetic DN-susceptible and DN-resistant mice that were not different in control groups (Table 1). Pathway analysis of this set of 29 genes again identified the differential regulation of the immune system as a significant feature of this comparison. Of the processes identified, ranked by lowest p-value, the top five were related to inflammation or the immune system (Table 2). Thus, in glomeruli, the immunologic response to diabetes, rather than background differences in non-immune system pathways, was significantly different between these mouse strains. The heat map for this comparison of 29 significantly different genes showing expression in each separate mouse, relative to the mean signal for diabetic DN-resistant mice, is depicted in Fig 3A.

**Table 1:** Significantly up- and down-regulated genes only in diabetic DN-susceptible vs.DN-resistant mice.

Up-regulated Genes		Down-regulated Genes	
Gene Name	Fold Change	Gene Name	Fold Change
Mdk	5.64	1300014I06Rik	0.50
Cyp4a12a	4.22	Csrnp1	0.44
Cyp4a12b	3.28	Tsc22d3	0.41
Sectm1b	2.90	Hmgn3	0.41
Hsd3b2	2.89	Rasl11b	0.38
Slc7a7	2.60	Cd300lg	0.36
Tmem82	2.41	Esm1	0.31
Lyplal1	2.35		
Fam20b	2.32		
Irgm1	2.32		
Adi1	2.29		
Plau	2.26		
C3	2.26		
Pde1a	2.22		
Dpp7	2.22		
Oas1g	2.19		
Prcp	2.19		
Kynu	2.19		
Ugt1a6a	2.13		
Ccbl2	2.08		
Apol9b	2.06		
Gabrb3	2.05		

**Table 2:** Pathway analysis of up- and down-regulated pathways only in diabetic DN-susceptible mice vs. DN-resistant mice.

Network	P-value	Min FDR*
Immune response_Lectin induced complement pathway	8.87E-10	2.34E-08
Immune response_Classical complement pathway	1.28E-09	2.34E-08
Immune response_Alternative complement pathway	1.28E-09	2.34E-08
Alternative complement cascade disruption in age-related macular degeneration	5.67E-07	7.80E-06
Complement pathway disruption in thrombotic microangiopathy	8.42E-05	9.26E-04
L-Methionine metabolism	2.91E-04	2.67E-03
Cortisol biosynthesis from Cholesterol	3.20E-03	2.51E-02
Cortisone biosynthesis and metabolism	5.70E-03	3.62E-02
Immune response_Antiviral actions of Interferons	5.92E-03	3.62E-02
Androstenedione and testosterone biosynthesis and metabolism p.1	6.83E-03	3.76E-02
Immune response_IL-4-induced regulators of cell growth, survival, differentiation and metabolism	8.59E-03	4.29E-02
Immune response_IFN-alpha/beta signaling via MAPKs	1.26E-02	5.78E-02
Androgen biosynthetic pathways	1.42E-02	6.02E-02
Immune response_Regulatory role of C1q in platelet activation	3.30E-02	1.30E-01

*, Min FDR, Minimum false discovery rate.

In addition to enrichment for immune-related genes, we noted three differentially expressed genes, in diabetic but not control DN-susceptible vs. DN-resistance mice, that were not originally classified in the pathway analysis as regulators of the immune system: Esm-1, Tsc22d3, and Midkine[24–26]. The enrichment analysis does not account for directionality. We noted that among these three gene transcripts associated in the literature with inhibition of leukocyte infiltration, expression of Esm-1 and Tsc22d3[24, 25] were significantly lower in DN-susceptible mice. In contrast, a gene transcript associated with promotion of leukocyte infiltration and more severe DN[26], Midkine, was higher in DN-susceptible mice. We validated that these results were consistent between microarray and qPCR experiments (Fig 3B–3D, S9 Table), using distinct primer sites to target the mRNA sequence. To begin to explore these differentially regulated genes with potential significance for DN, we selected Esm-1 for further characterization. Of note, we also extracted RNAseq data from the Nephroseq online resource[27] comparing glomerular expression data from healthy donors vs. individuals with DN. In this cohort, consistent with our data, individuals with DN had significantly lower Esm-1 (S6 Fig).

### High expression of Esm-1 and strain-specific difference is found in glomeruli

**Fig 4:**
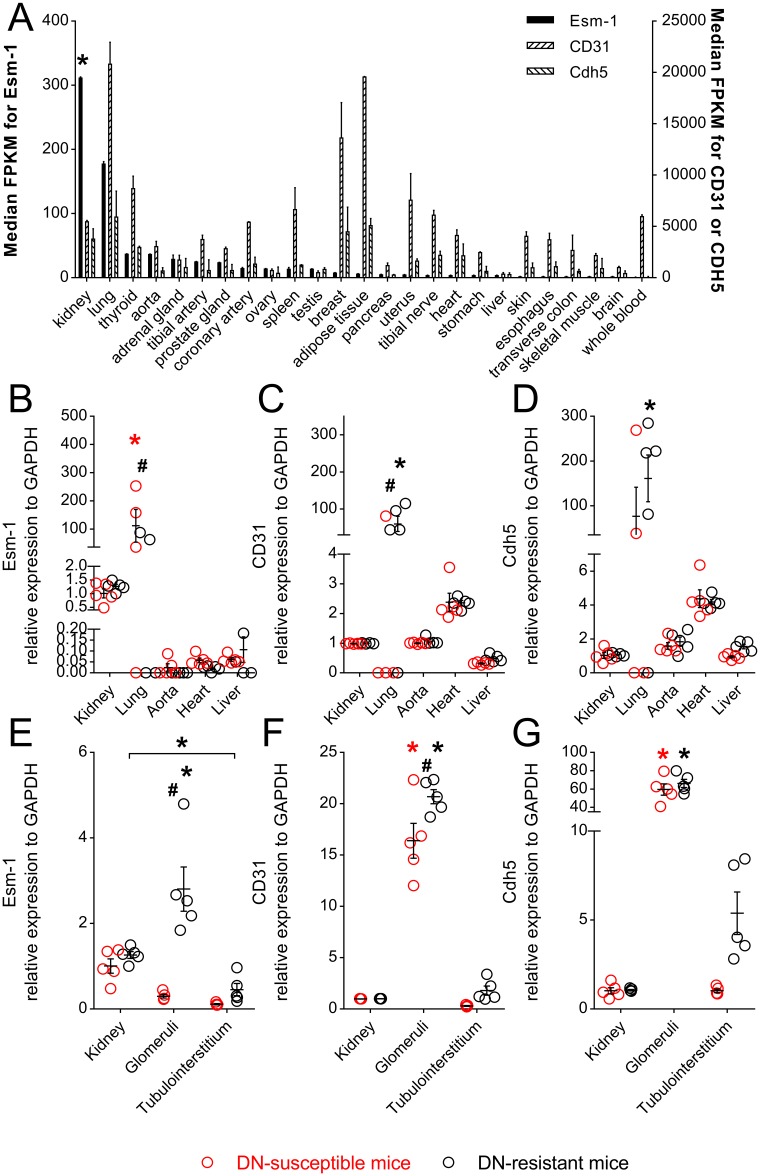
Esm-1 is high in human and mouse kidney and selectively enriched in DN-resistant mice glomeruli. (A) Median Fragments Per Kilobase of transcript per Million mapped reads of Esm-1 (*black*), CD31 (*left hatched*), and Cadherin 5 (Cdh5, *right hatched*) mRNA from seven human tissue RNAseq datasets are shown. Kidney and lung have significantly higher Esm-1 expression than thyroid, aorta, adrenal gland, and tibial artery, and this pattern does not parallel two markers of endothelial cells, CD31 and Cdh5. *, p-value < 0.05 vs. thyroid, aorta, adrenal gland, and tibial artery. (B-G) Quantitative PCR for Esm-1, CD31, and Cdh5 mRNA from select tissues (B-D) and kidney compartments (E-G) is shown. Kidney samples from control, DN-resistant mice were used as the reference group. Each open circle represents data from one mouse. Red and black circles/asterisks indicate data from DN-susceptible and DN-resistant mice, respectively. *, p-value < 0.05 in the same mouse strain between different tissues/compartments; #, p-value < 0.05 in the same tissue/compartment between the two mouse strains. N = 4–5 mice per group; n = 3 replicate wells per sample.

To ascertain whether Esm-1 was ubiquitously or selectively expressed, we surveyed tissue-specific expression in both humans and mice. We compared Esm-1 expression in datasets from several experiments involving 25 human tissues, and kidney Esm-1 mRNA is the highest, followed by lung (Fig 4A). This difference was not driven by differences in endothelial cell number as two markers for endothelial cells, CD31 and Cadherin 5[28], are not similarly expressed across human tissues. We also compared Esm-1 expression in several tissues from both DN-susceptible and DN-resistant mouse strains, and similar to humans, Esm-1 expression in kidney and lung is highest (Fig 4B). As in humans, this difference was not likely due to a difference in endothelial cell number as CD31 and Cadherin 5 are not similarly expressed across tissues (Fig 4C and 4D) [28]. Within kidney, Esm-1 expression is significantly enriched for in glomeruli, and is only significantly lower in DN-susceptible vs. resistant mice within this compartment (0.30 ± 0.09 vs. 2.81 ±1.12, p<0.05) (Fig 4E–4G). We next examined the regulation of Esm-1 within glomeruli.

### Glomerular Esm-1 secretion inversely correlates with DN susceptibility

**Fig 5:**
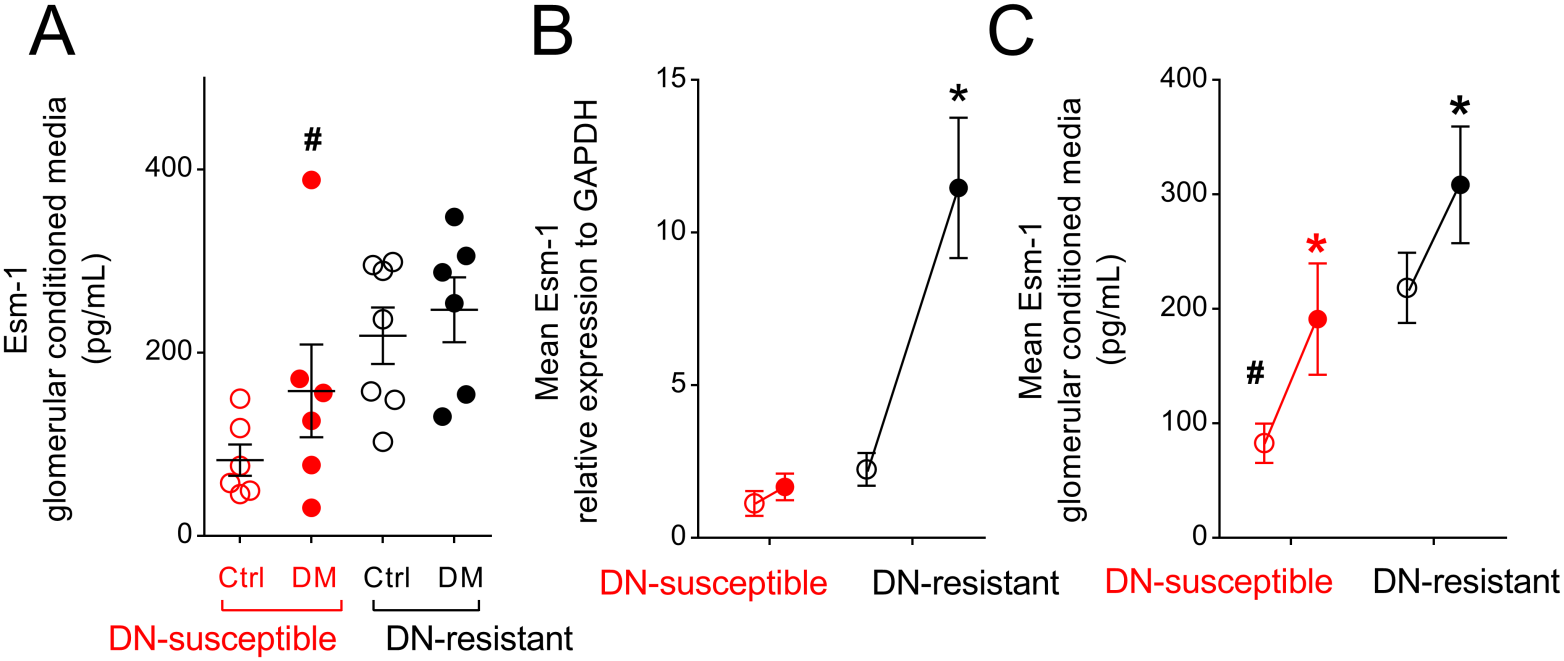
Attenuated secretion of glomerular Esm-1 with diabetes/high glucose in DN-susceptible compared to DN-resistant mice. (A) Glomeruli were isolated from mice treated with vehicle or streptozotocin for 4 weeks and cultured for 24 hours. Mouse Esm-1 was measured by ELISA from conditioned media. Each circle represents data from one mouse. Open and closed circles indicate data from vehicle-injected and streptozotocin-injected (i.e. control and diabetic) mice, respectively. Ctrl, control; DM, diabetic. (B-C) Glomeruli were isolated from mice treated with vehicle only and cultured in low (100 mg/dL) or high glucose (450 mg/dL) for 24 hours. Mean Esm-1 mRNA (B) and secreted protein (C) were compared between low (open circles) and high glucose (closed circles) pairwise from individual mouse glomeruli. Each circle represents the group mean. Red and black circles/asterisks indicate data from DN-susceptible and DN-resistant mice, respectively. *, p-value < 0.05 in the same mouse strain between control and diabetic or between low and high glucose media groups; #, p-value < 0.05 in the same treatment between the two mouse strains. N = 6–7 mice per group; n = 3 replicate wells per sample in qPCR, and n = 2 replicate wells per sample in ELISA.

Esm-1 is predominantly secreted[22], and we were unable to detect Esm-1 in glomerular lysates by Western blot analysis. Therefore, to test whether Esm-1 protein expression in glomeruli is regulated by diabetes, we isolated and cultured glomeruli, and assayed for Esm-1 in conditioned media by ELISA. Similar to mRNA expression, four weeks after streptozotocin injection, glomeruli from DN-susceptible mice secreted significantly less Esm-1 than DN-resistant mice (Fig 5A).

### High glucose concentration increases glomerular-derived Esm-1

To test whether glucose directly stimulates local glomerular Esm-1 secretion, we isolated glomeruli from control DN-susceptible and DN-resistant mice, and assayed for Esm-1 mRNA and protein secretion in low or high glucose media. Incubation in high glucose media increased Esm-1 mRNA and protein secretion in glomeruli from both strains of mice (Fig 5B and 5C), however the increase was significantly less in DN-susceptible mice.

### Systemic Esm-1 is dynamically regulated in diabetes

**Fig 6:**
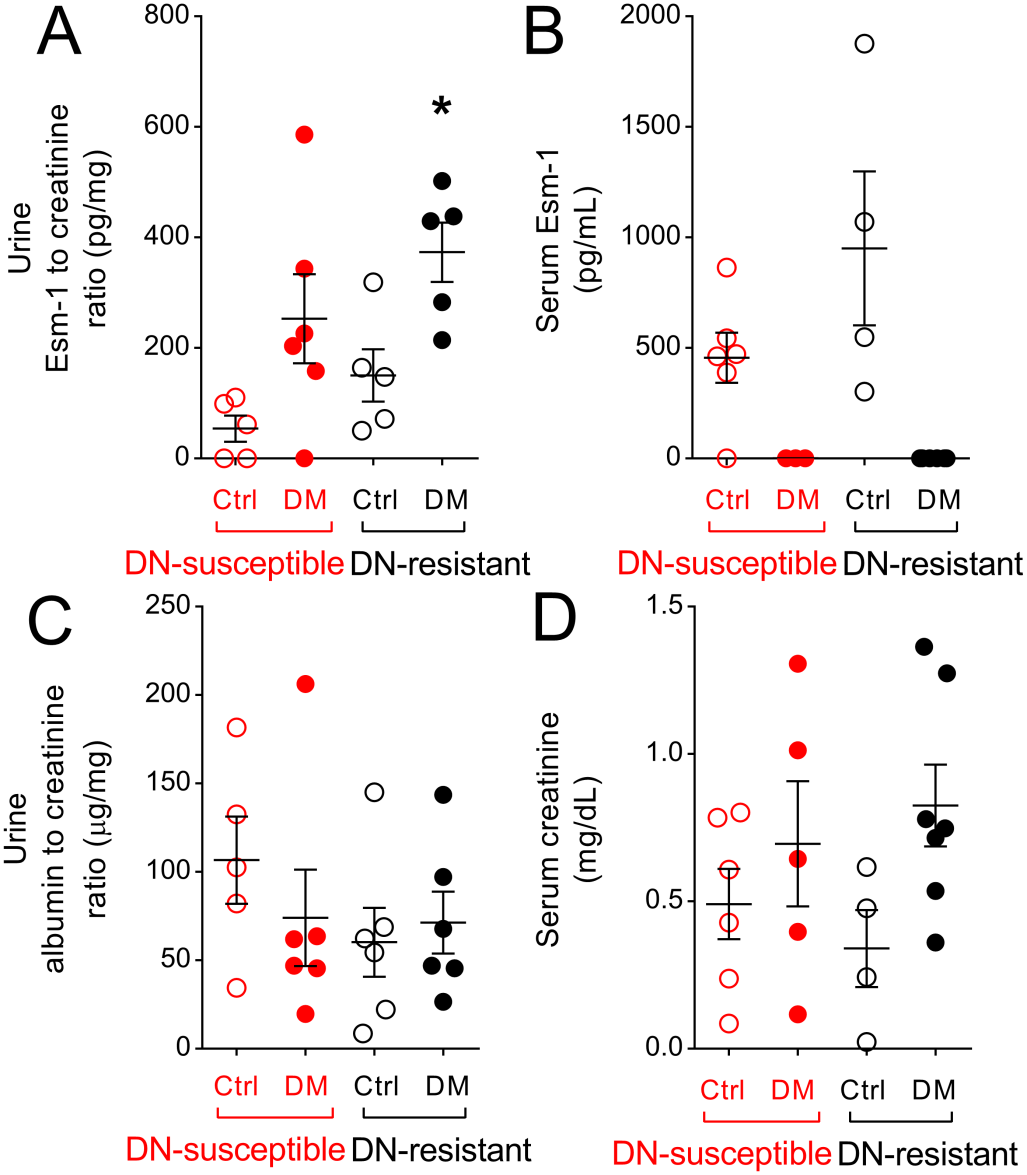
Urine Esm-1 is increased in diabetic mice. Esm-1 level was measured in urine (A) and serum (B) from control and diabetic mice. Urine albumin-to-creatinine ratio (C), a marker of glomerular permeability, and serum creatinine (D), a surrogate for glomerular filtration rate from control and diabetic mice are shown. Each circle represents data from one mouse. Open and closed circles indicate data from vehicle- and streptozotocin-injected (i.e. control and diabetic) mice, respectively. Red and black circles/asterisks indicate data from DN-susceptible and DN-resistant mice, respectively. Ctrl, control; DM, diabetic; *, p-value < 0.05 in the same mice strain between control and diabetic groups; N = 3–7 mice per group; n = 2 replicate wells per sample.

The glomerulus is a major source of Esm-1 production (S3 Fig), but the contribution of kidney production to urine or serum Esm-1 has never been tested. We measured urine and serum Esm-1 from control and diabetic mice after 4 weeks of vehicle or streptozotocin injection, respectively. Urine Esm-1 was significantly higher in DN-resistant mice (Fig 6A). To determine the contribution of circulating Esm-1, we measured serum Esm-1 in similar groups of mice (Fig 6B). In contrast to urine levels, diabetes significantly decreased circulating Esm-1. To test the integrity of the glomerular filtration barrier, we measured the urine albumin-to-creatinine ratio, and at 4 weeks after streptozotocin or vehicle injection, the ratio was similar among diabetic and control mice from both strains (Fig 6C). Serum creatinine, a surrogate marker for glomerular filtration, was also similar among all groups (Fig 6D).

### Esm-1 inhibits leukocyte transmigration in a dose-dependent manner

**Fig 7:**
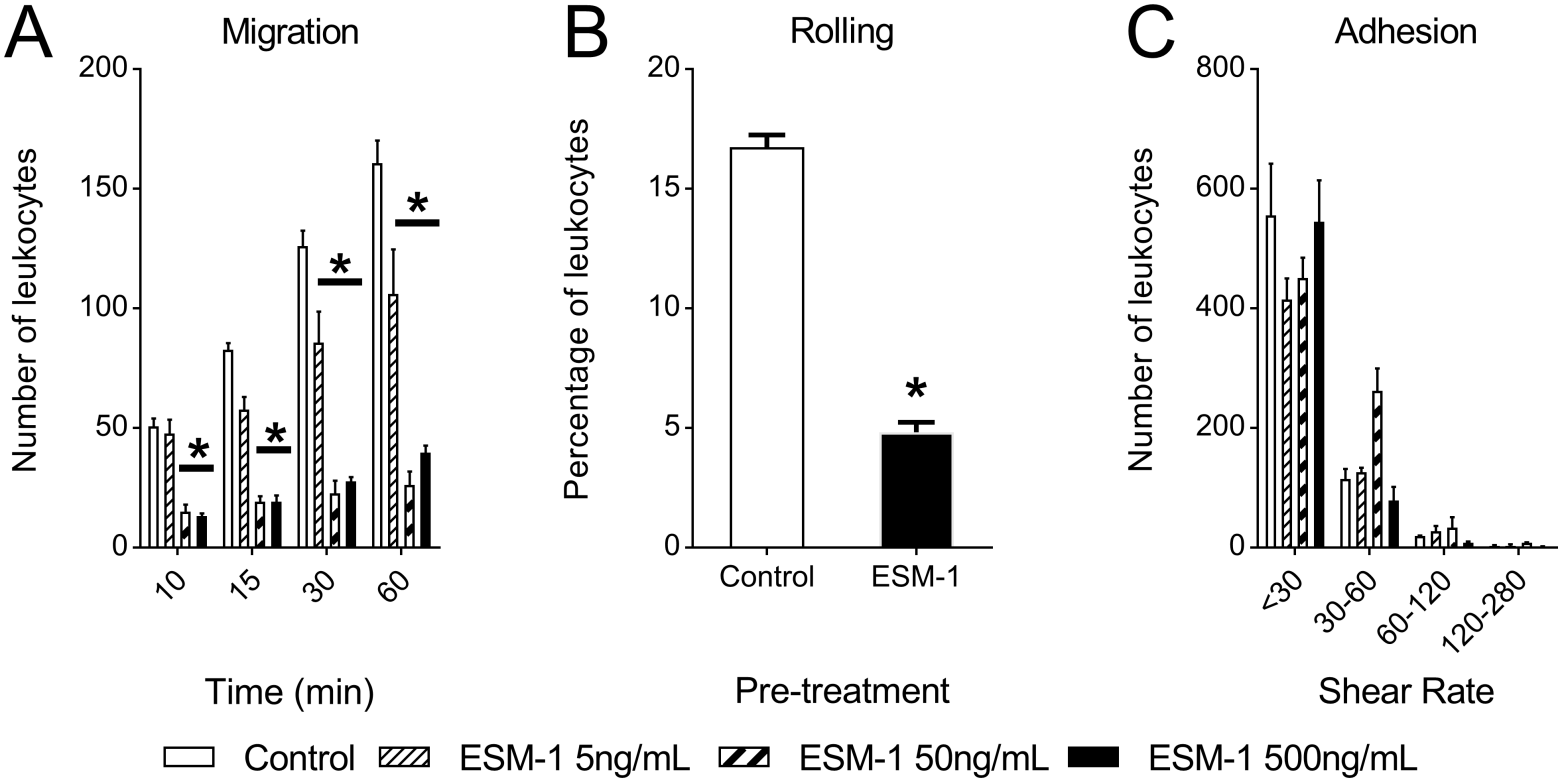
Pre-treatment with Esm-1 inhibits leukocyte infiltration in a dose-dependent manner. A three-dimensional monolayer of endothelial cells is seeded on vessels of a biomimetic microfluidic assay (bMFA) and an activated neutrophil suspension pre-incubated with or without increasing doses of recombinant Esm-1 is injected into the bMFA vascular network. (A) The number of neutrophils transmigrating across endothelial cells at indicated time intervals is quantified relative to untreated neutrophils. (B) The percentage of leukocytes rolling after pre-treatment with vehicle (control) or recombinant Esm-1 is shown. (C) The number of adherent neutrophils on the microfluidic chip is depicted at indicated shear rates. N = 3 experiments per dose. *, p-value < 0.05 vs. vehicle group.

To test directly whether Esm-1 blocks leukocyte infiltration across an endothelial monolayer, we utilized intravital microscopy and a biomimetic microfluidic assay (bMFA). Pre-treatment of leukocytes with recombinant Esm-1 showed significantly decreased transmigration (Fig 7A) at 30 and 60 minutes in a dose-dependent manner, suggesting an inhibitory role of Esm-1 against leukocyte infiltration in DN. To investigate the mechanism of decreased transmigration, we examined the role of recombinant Esm-1 to inhibit leukocyte rolling and adhesion (Fig 7B and 7C). In this *ex vivo* assay Esm-1 did not influence leukocyte adhesion. However, leukocyte rolling was significantly decreased in the presence of recombinant Esm-1 vs. vehicle.

## Discussion

Our comparisons of control vs. diabetic glomerular RNA from both DN-susceptible and DN-resistant mice demonstrate differences in transcripts related to the immune system. While inflammation is a known component of DN[29–33], we discovered that early expression of glomerular genes related to inflammation is a differentiating marker of susceptibility to DN. Hodgin et al. first demonstrated that differences in glomerular gene transcripts correlate with differences in severity of DN[34]. However, these differences were examined at a late stage of disease, and importantly, compared the response to diabetes in DN-susceptible groups but not the genetic background between susceptible and resistant groups. Based on seminal studies by the Diabetic Complications Consortium [15, 16], we chose DBA/2 and C57BL/6 mice for comparison. Additionally, allele-specific gene sequencing from F1 progeny of these two strains reveals that 41% of genes are differentially expressed in at least one tissue[35]. At an early stage of disease with similar levels of hyperglycemia and when histologic and clinical indices of DN were not yet present[15, 16], DN-susceptible mice have more glomerular leukocyte infiltration compared with DN-resistant mice[17], and our data suggest that leukocyte infiltration is due, in part, to glomerular-specific changes in expression rather than systemic determinants of inflammation.

We sought to identify *glomerular-derived* regulators of leukocyte infiltration as these have not been well characterized. Several lines of evidence suggest that leukocyte infiltration may contribute substantially to glomerular injury, fibrosis, and albuminuria in DN[11, 36–39]. First, glomerular and tubulointerstitial infiltration of macrophages is observed in the diabetic kidney from mice and humans[17, 32, 36, 38], and is proportional to the level of albuminuria[32]. Second, genetic deletion of inflammatory mediators, e.g., leukocyte-attracting chemokine, Monocyte chemotactic protein 1, or intercellular adhesion molecule, ICAM-1[37–39], attenuates the progression of DN in mice. More recently, macrophage-derived factors aggravate glomerular endothelial damage in DN in mice[17]. However, drugs that block leukocyte-endothelial interaction in multiple tissues, e.g., efaluzimab[40], have been removed from the market due to life-threatening infections, highlighting the need for more tissue-specific targeting of the delicate interaction between leukocytes and endothelial cells.

Esm-1 is highly expressed in kidney glomeruli. This relatively high kidney expression was not based on endothelial number, and in fact, the relative difference between mouse kidney and lung could be accounted for by higher levels of endothelial cell markers in lung. However, within mouse kidney, glomeruli had the highest Esm-1 expression likely due to significant enrichment of endothelial cells in this compartment relative to the tubulointerstitium. Interestingly, the strain-specific deficiency of glomerular Esm-1 in DN-susceptible mice was not observed in other tissues. Therefore, we speculate that other glomerular-derived genes may influence Esm-1 transcription rather than a more systemic difference (e.g., Esm-1 promoter sequence) between these genetically distinct strains. Furthermore, glomerular Esm-1 is enriched in endothelial cells[41], consistent with a gene that is native to glomeruli rather than derived from an infiltrating cell. Moreover, lower Esm-1 expression in DN-susceptible mice would be unlikely due to podocytopenia[20]. We further explored the regulation and function of Esm-1 both *in vivo* and *ex vivo*.

Several pro-inflammatory mediators (e.g. TNFα) can induce expression of Esm-1[42], but heretofore, the direct influence of the diabetic milieu on Esm-1 expression was unknown. Interestingly, this attenuated increase in a susceptible cohort in a *chronic* disease is reminiscent of serum Esm-1 levels in patients with *acute* lung injury, i.e., inflammatory mediators that accompany sepsis can induce Esm-1, but the patients with higher mortality, presumably due to more inflammation, had a smaller increase in Esm-1[43]. The relative deficiency of secreted glomerular Esm-1 in DN-susceptible mice is congruent with the mRNA data but the magnitude of the difference is attenuated. This could be due to alterations in glomerular endothelial cells due to isolation and plating of the glomeruli. We found that after 24 hours, Esm-1 secretion decreased dramatically in culture (S3 Fig). Perhaps earlier time points would demonstrate a larger difference between strains of mice, but the detection limit of the ELISA precluded this analysis. Differences in secretion between strains may also be influenced by differences in trafficking of Esm-1 to the plasma membrane or degradation of secreted Esm-1.

To dissect the mechanisms that regulate Esm-1, we demonstrated that high glucose is sufficient to increase glomerular Esm-1 mRNA and protein *in vitro*, and significantly more in DN-resistant compared to DN-susceptible mice. Cytokines that stimulate Esm-1 mRNA (e.g. VEGF) possibly mediate the effect of high glucose as these cytokines are also acutely regulated by high glucose in cultured mesangial cells[44, 45]; however, whether these cytokines participate in DN susceptibility remains an area of further study. Esm-1 transcription is negatively regulated by the transcription factor hHex, but this gene was not differentially expressed in our microarray analysis[46]. Future studies will explore the effects of high glucose on hHex or its binding sites within the Esm-1 promoter and on mechanisms of differential transcription (e.g. promoter methylation) and processing of Esm-1 in glomeruli.

To explore whether this differential expression of kidney Esm-1 is reflected *in vivo*, we measured urine and serum Esm-1. In DN-resistant, compared to DN-susceptible mice, urine Esm-1 was significantly increased with diabetes. Conversely, serum Esm-1 was surprisingly decreased with diabetes. Moreover, a marker of glomerular membrane permeability, urine albumin-to-creatinine ratio, remained unchanged 4 weeks after induction of diabetes. Thus, the filtered load of Esm-1 is not expected to increase. These results suggest that urine Esm-1 may be a candidate non-invasive biomarker of glomerular Esm-1, which increases with hyperglycemia and diabetes and correlates directly with DN resistance. Urine Esm-1 could possibly reflect Esm-1 secretion into the tubule by endothelial cells along the vasa recta. However, tubular secretion would imply transepithelial or paracellular transport of a large ~50kDa protein which is less likely. The mechanism of decreased serum Esm-1 in diabetes is unknown. The diabetic milieu may induce an Esm-1-directed serum protease. The disconnect between urine and serum Esm-1 also suggests that glomeruli may not contribute significantly to circulating Esm-1. Thus, urine Esm-1 is a potential non-invasive biomarker of glomerular Esm-1 production and protection from leukocyte infiltration.

We further characterized the ability of prevent leukocyte recruitment. Esm-1 binds activated leukocyte free antigen-1 (LFA-1), and antagonizes interaction with endothelial-cell expressed ICAM-1[42] in a dose-dependent manner *in vitro*[24]. By using a biomimetic microfluidic assay which includes a vascular network in communication with a tissue compartment to mimic physiological flow conditions[23, 47] we found that recombinant Esm-1 was sufficient to directly inhibit rolling and transmigration *ex vivo*. The lowest, significant dose for inhibition of leukocyte transmigration (5 ng/mL) is within the physiologic range of glomerular-secreted Esm-1 if we assume that the volume of an isolated Bowman’s capsule is 1.5 x10^5^ μm^3^ [48]. Surprisingly, leukocyte-to-endothelial cell adhesion was not reduced by Esm-1. These data would suggest that LFA-1 is not the only adhesion molecule on leukocytes that binds to ICAM-1[49]. Moreover, these data also underscore the importance of studying leukocyte infiltration in a microfluidic assay, where *in vivo* flow conditions can be modeled[50], and the direct influence on specific mechanisms of leukocyte infiltration (i.e., an effect on rolling, adhesion, and/or migration) can be studied in a vascular network. Our assay was optimized with human neutrophils and human umbilical vascular endothelial cells, rather than macrophages and glomerular endothelial cells, and the role of Esm-1 in leukocyte subtypes and tissue- and species-specific endothelial cells should be confirmed. Moreover, the mechanisms for how Esm-1 decreases rolling remain unexplored.

To our knowledge, this is the first detailed characterization of Esm-1 in diabetes and in kidney with respect to inflammation and DN susceptibility. Consistent with our data, the Nephroseq online database demonstrates a lower expression of glomerular Esm-1 in individuals with DN vs. healthy human donors, and serum Esm-1 is lower in individuals with diabetes mellitus (1.75 ± 0.78 ng/mL) vs. healthy controls (2.20 ± 0.54 ng/mL)[51], although the susceptibility to DN is not known. Esm-1 is enriched in endothelial cells from glomeruli over whole kidney and was higher in glomeruli from *db/db* mice vs. controls by IHC[41]. It is conceivable that the *db/db* mice used in that study was derived from C57BL/6 vs. KS background (which shares ~14% of the DBA/2, DN-susceptible genome)[19]. Esm-1 was also proportional to markers of inflammation in a cohort of patients with chronic kidney disease[52]. Based on the functional role of Esm-1 that we demonstrated in this study, Esm-1 levels in this prior study may not be an initiator of morbidity but rather a compensatory signal to combat inflammation.

The implications of our work may also extend to acute states of glomerular injury. Glomerular Esm-1 mRNA is decreased in LPS- and anti-GBM-treated mice compared to respective controls[53, 54]. Similar to DN, these acute injury models are ICAM-1 dependent[55], and thus the contribution of a glomerular-derived inhibitor of acute inflammation will be explored in future studies. This strategy is particularly appealing for the pulmonary-renal syndrome of anti-GBM disease[56, 57] as Esm-1 is primarily expressed in kidney but also lung.

In summary, our unbiased screen of glomerular-derived transcripts from DN-susceptible and DN-resistant mice, uncovers Esm-1 as a potential protective gene. This protein is up-regulated in glomeruli and in urine from diabetic vs. control mice, and correlates with resistance to DN. Moreover, *ex vivo* on-chip studies suggest a role for Esm-1 as an inhibitor of leukocyte transmigration across endothelium. These findings motivate further studies of the role of Esm-1 in protecting against DN *in vivo*, and as a marker of resistance to glomerular inflammation in acute and chronic kidney diseases.

## Supporting information

S1 Fig Quality control of the purity of isolated glomeruli for microarray experiments. cDNA was prepared from isolated glomeruli and whole kidney from both DN-susceptible and DN-resistant mice. Nephrin, a podocyte marker of glomeruli, was measured by real-time PCR. Kidney samples from DN-resistant mice are used as the reference group. Each circle represents data from one mouse. Red and black circles indicate data from DN-susceptible and DN-resistant mice, respectively. *, p-value < 0.05 in the same mouse strain between whole kidney and glomeruli. N = 3–4 mice per group; n = 3 replicated wells per sample.
(TIF)

S2 Fig Purity of glomerular and tubulointerstitial fractions is shown by nephrin and SGLT2 expression, respectively. cDNA was prepared from isolated glomeruli and tubulointerstitial fractions from both DN-susceptible and DN-resistant mice. qPCR was performed to quantify nephrin expression. Kidney samples from DN-resistant mice are used as the reference group. Each circle represents data from one mouse. Red and black circles indicate data from DN-susceptible and resistant mice, respectively. *, p-value < 0.05 in the same mice strain between whole kidney and glomerular or tubulointerstitial fractions; #, p-value < 0.05 in the same treatment between the two mice strains. N = 4–5 mice per group; n = 3 replicated wells per sample.
(TIF)

S3 Fig Mouse ELISA is specific for Esm-1 and is sufficiently sensitive to detect secreted glomerular Esm-1. (A) Positive controls: the conditioned media of HEK293T cells transfected with mouse Esm-1-expressing plasmid (mEsm-1), and cell lysate containing mouse Esm-1 provided by the manufacturer (Kit positive control). Negative controls: the conditioned media of 293T cells transfected with human Esm-1-expressing plasmid (hEsm-1) or empty plasmid. (B) Glomeruli were isolated from DN-resistant, C57BL/6 mice and cultured in DMEM-0.2%FCS for 24 hours, 48 hours and 2 weeks. Mouse Esm-1 was measured by ELISA using conditioned media. Media not exposed to glomeruli was used as negative control. n = 2 replicated wells per sample.
(TIF)

S4 Fig DN-susceptible and DN-resistant mice have sustained hyperglycemia after four weeks. Eight week-old DN-susceptible and DN-resistant mice were injected with streptozotocin (STZ, 45–50 mg/kg body weight) vs. vehicle for 5 consecutive days. Hyperglycemia was validated by fasting blood glucose 4 weeks after STZ. Open and closed circles indicate data from vehicle- and STZ-injected (i.e. control and diabetic) mice, respectively. Red and black circles indicate data from DN-susceptible and resistant mice, respectively. *, p-value < 0.05 in the same mouse strain between control and diabetic groups. #, p-value < 0.05 in the same treatment between the two mouse strains. Ctrl, control; DM, diabetic; N = 4 mice per group; n = 2 replicated wells per urine ELISA sample.
(TIF)

S5 Fig DN-susceptible and DN-resistant mice have significantly different levels of albuminuria. Eight week-old DN-susceptible and DN-resistant mice were injected with streptozotocin (STZ, 45–50 mg/kg body weight) vs. vehicle for 5 consecutive days. To validate differential susceptibility after long-standing diabetes, urine albumin-to-creatinine ratio was measured 16 weeks after STZ in a separate group of mice. Open and closed circles indicate data from vehicle- and STZ-injected (i.e. control and diabetic) mice, respectively. Red and black circles indicate data from DN-susceptible and resistant mice, respectively. *, p-value < 0.05 in the same mouse strain between control and diabetic groups. #, p-value < 0.05 in the same treatment between the two mouse strains. Ctrl, control; DM, diabetic; N = 3–5 mice per group; n = 2 replicated wells per urine ELISA sample.
(TIF)

S6 Fig Esm-1 expression level is lower in individuals with DN. RNAseq data of Esm-1 is compared from the Nephroseq online resource. Open and closed circles indicate data from healthy human donors and individuals with DN, respectively. * p-value < 0.05. N = 9–13 individuals per group.
(TIF)

S1 Methods Transfection of HEK293T cells and expression data in Nephroseq. (DOCX)

S1 Table Significantly differentially expressed genes in control vs. diabetic DN-susceptible mice. (DOCX)

S2 Table Pathway analysis of up- and down-regulated pathways in control vs. diabetic DN-susceptible mice. (DOCX)

S3 Table Significantly differentially expressed genes in control vs. diabetic DN-resistant mice. (DOCX)

S4 Table Pathway analysis of up- and down-regulated pathways in control vs. diabetic DN-resistant mice. (DOCX)

S5 Table Significantly differentially expressed genes in control DN-susceptible vs. DN-resistant mice. (DOCX)

S6 Table Pathway analysis of up- and down-regulated pathways in control DN-susceptible vs. DN-resistant mice. (DOCX)

S7 Table Significantly differentially expressed genes in diabetic DN-susceptible vs. DN-resistant mice. (DOCX)

S8 Table Pathway analysis of up- and down-regulated pathways in diabetic DN-susceptible vs. DN-resistant mice. (DOCX)

S9 Table Comparing expression by Microarray and qPCR. (DOCX)

## Abbreviations

bMFA: Biomimetic microfluidic assay
C57BL/6: C57 black 6
DBA/2: Dilute brown non-agouti
DN: Diabetic nephropathy
Esm-1: Endothelial cell-specific molecule-1
GBM: Glomerular basement membrane
Hhex: Hematopoietically expressed homeobox
LFA-1: Leukocyte free antigen 1
qPCR: Quantitative PCR
RNAseq: RNA sequencing
Tsc22d3: Tsc22 domain family member 3
