# Supplementary figures and images for: Murine glomerular transcriptome links endothelial cell-specific molecule-1 deficiency with susceptibility to diabetic nephropathy
Source: PLoS One. 2017 Sep 21;12(9):e0185250. doi: 10.1371/journal.pone.0185250 (PMC5608371; doi:10.1371/journal.pone.0185250)

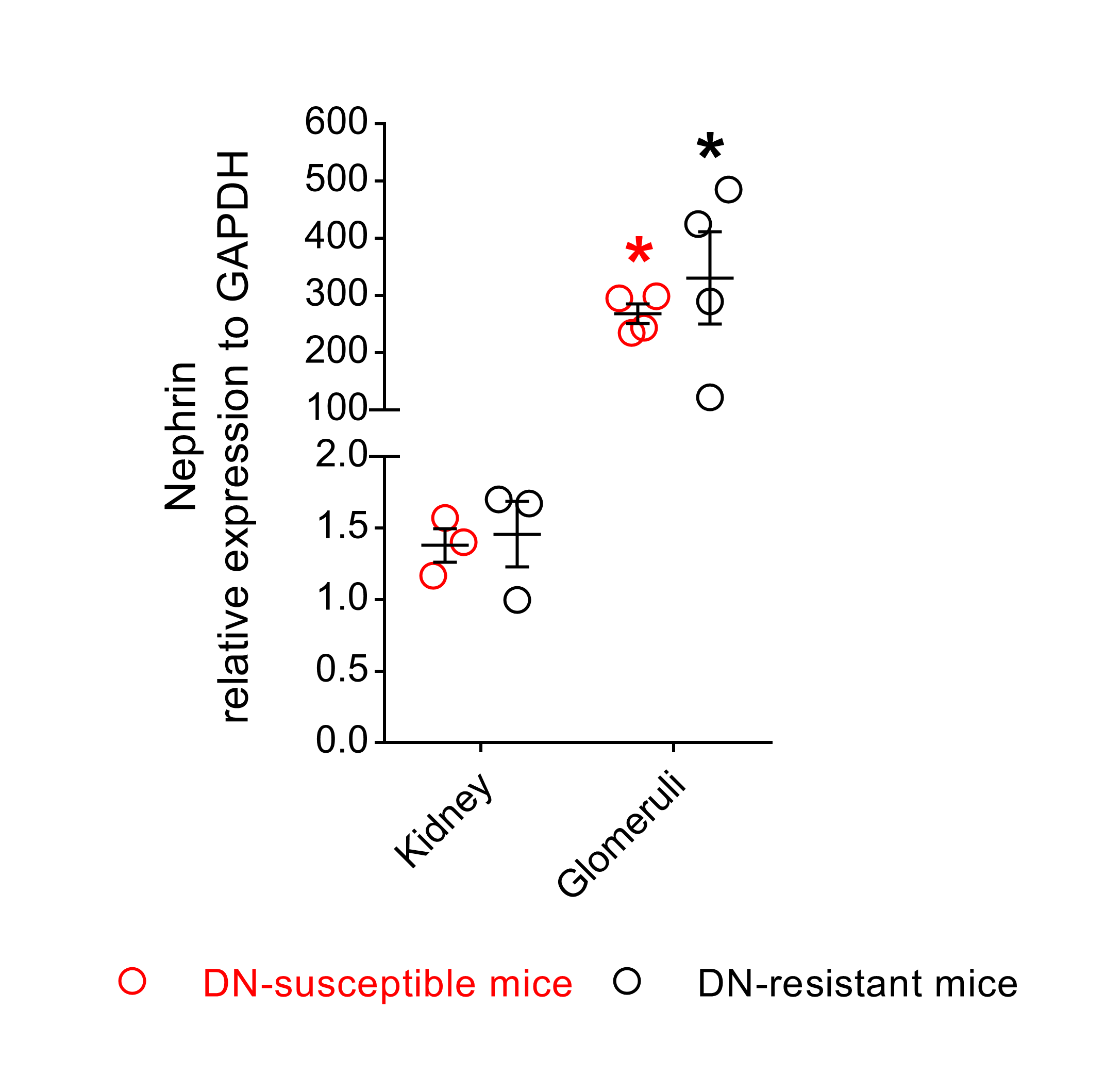

Supplement: S1 Fig — cDNA was prepared from isolated glomeruli and whole kidney from both DN-susceptible and DN-resistant mice. Nephrin, a podocyte marker of glomeruli, was measured by real-time PCR. Kidney samples from DN-resistant mice are used as the reference group. Each circle represents data from one mouse. Red and black circles indicate data from DN-susceptible and DN-resistant mice, respectively. *, p-value < 0.05 in the same mouse strain between whole kidney and glomeruli. N = 3–4 mice per group; n = 3 replicated wells per sample. (TIF) [file pone.0185250.s001.tif]

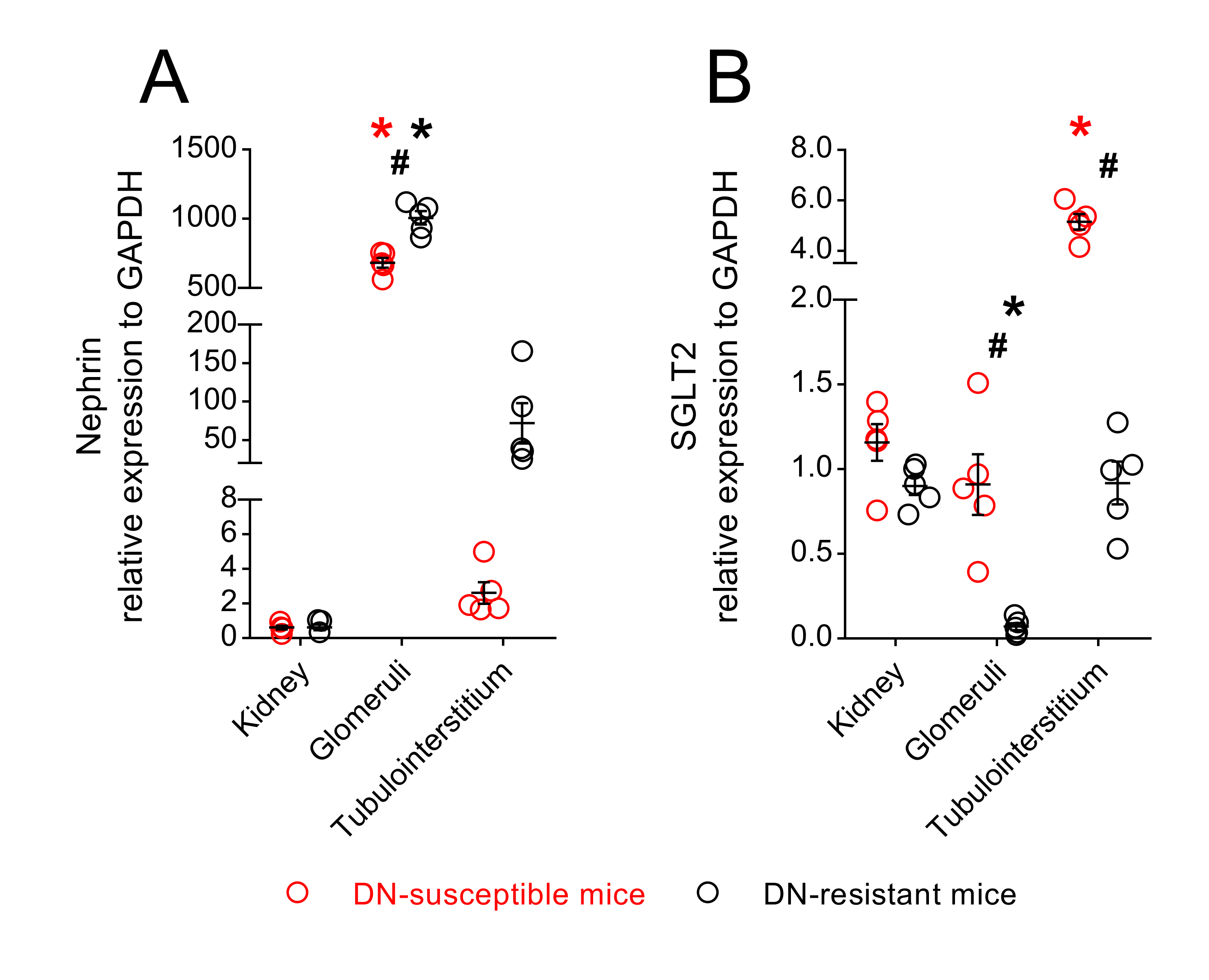

Supplement: S2 Fig — cDNA was prepared from isolated glomeruli and tubulointerstitial fractions from both DN-susceptible and DN-resistant mice. qPCR was performed to quantify nephrin expression. Kidney samples from DN-resistant mice are used as the reference group. Each circle represents data from one mouse. Red and black circles indicate data from DN-susceptible and resistant mice, respectively. *, p-value < 0.05 in the same mice strain between whole kidney and glomerular or tubulointerstitial fractions; #, p-value < 0.05 in the same treatment between the two mice strains. N = 4–5 mice per group; n = 3 replicated wells per sample. (TIF) [file pone.0185250.s002.tif]

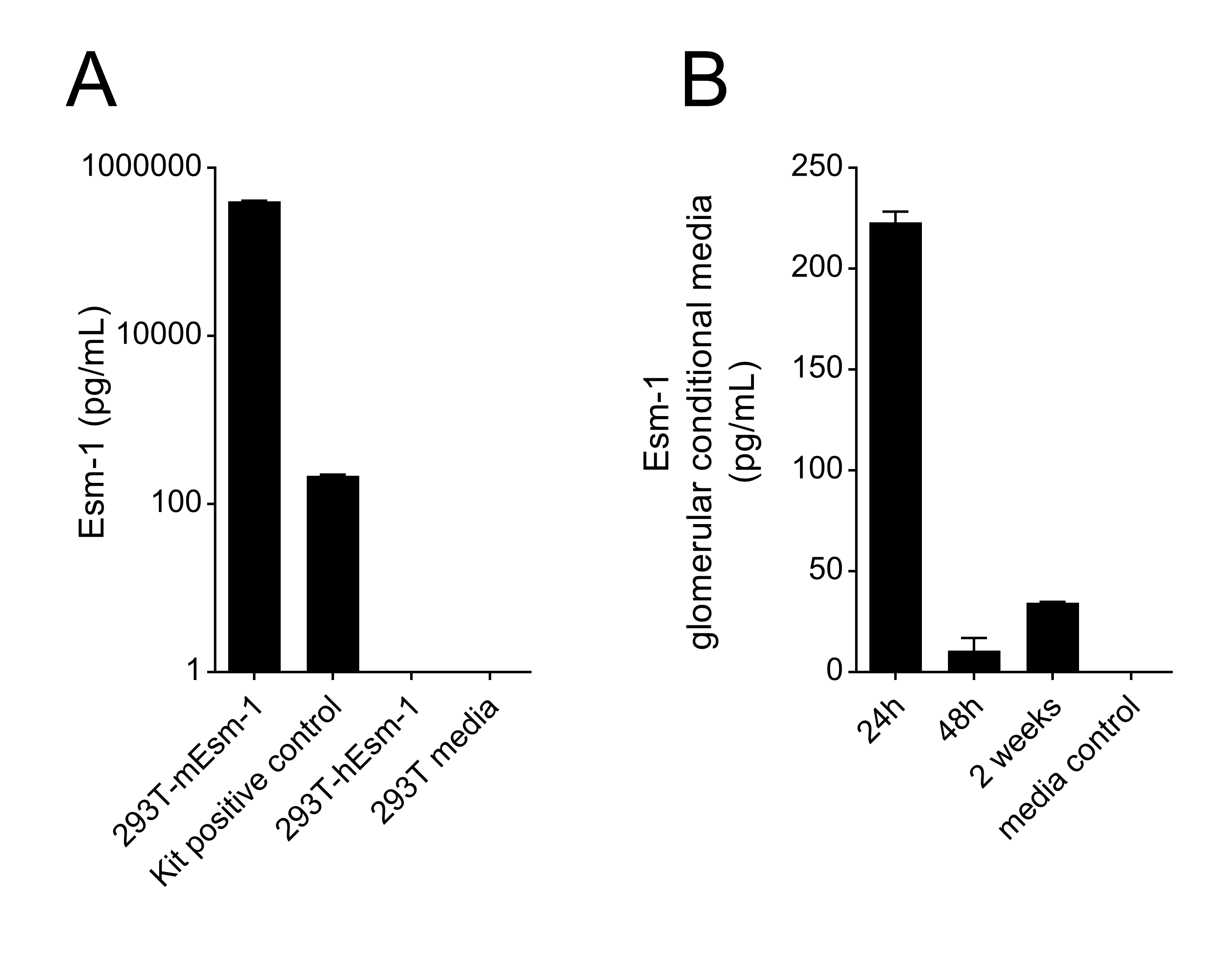

Supplement: S3 Fig — (A) Positive controls: the conditioned media of HEK293T cells transfected with mouse Esm-1-expressing plasmid (mEsm-1), and cell lysate containing mouse Esm-1 provided by the manufacturer (Kit positive control). Negative controls: the conditioned media of 293T cells transfected with human Esm-1-expressing plasmid (hEsm-1) or empty plasmid. (B) Glomeruli were isolated from DN-resistant, C57BL/6 mice and cultured in DMEM-0.2%FCS for 24 hours, 48 hours and 2 weeks. Mouse Esm-1 was measured by ELISA using conditioned media. Media not exposed to glomeruli was used as negative control. n = 2 replicated wells per sample. (TIF) [file pone.0185250.s003.tif]

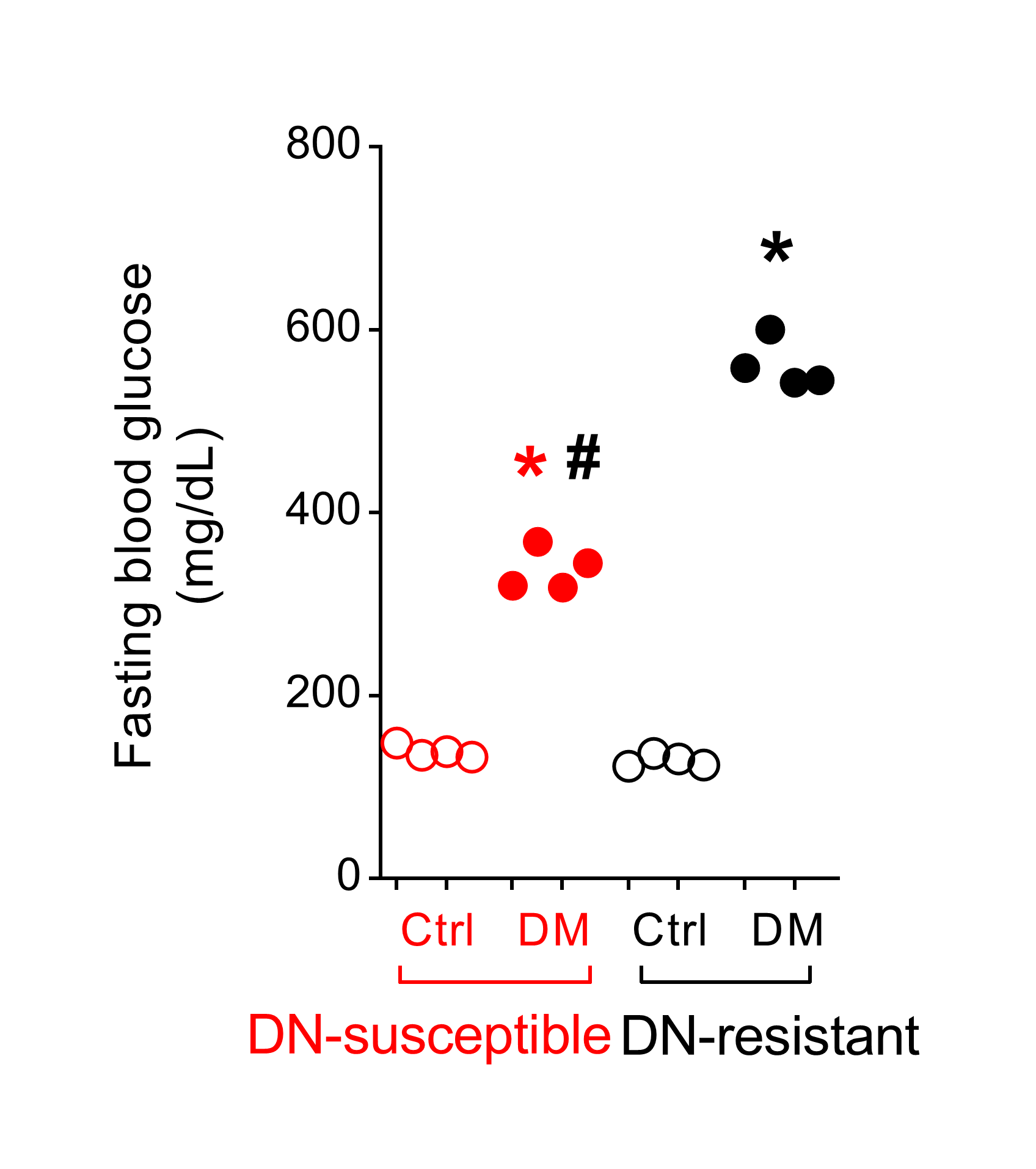

Supplement: S4 Fig — Eight week-old DN-susceptible and DN-resistant mice were injected with streptozotocin (STZ, 45–50 mg/kg body weight) vs. vehicle for 5 consecutive days. Hyperglycemia was validated by fasting blood glucose 4 weeks after STZ. Open and closed circles indicate data from vehicle- and STZ-injected (i.e. control and diabetic) mice, respectively. Red and black circles indicate data from DN-susceptible and resistant mice, respectively. *, p-value < 0.05 in the same mouse strain between control and diabetic groups. #, p-value < 0.05 in the same treatment between the two mouse strains. Ctrl, control; DM, diabetic; N = 4 mice per group; n = 2 replicated wells per urine ELISA sample. (TIF) [file pone.0185250.s004.tif]

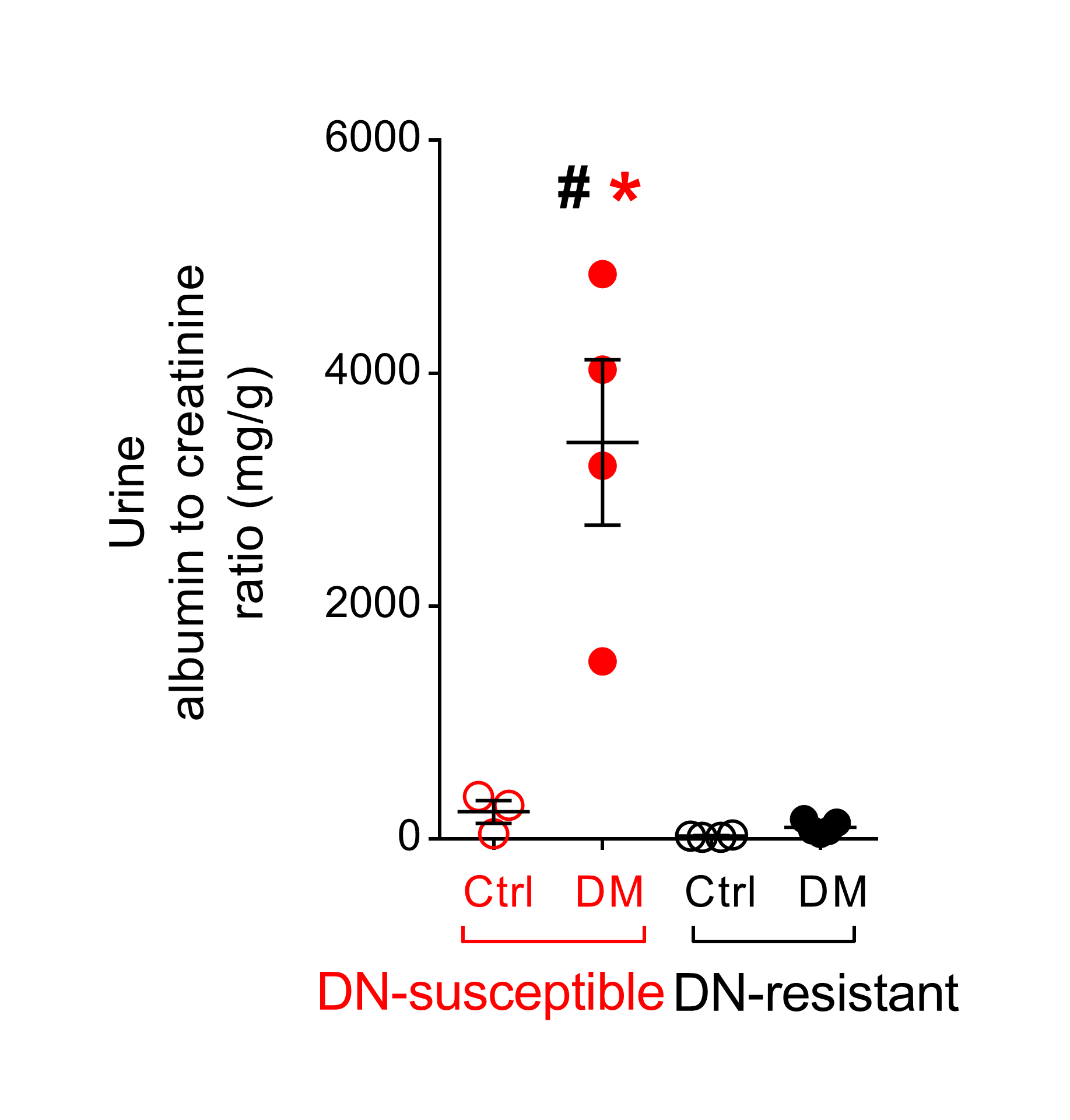

Supplement: S5 Fig — Eight week-old DN-susceptible and DN-resistant mice were injected with streptozotocin (STZ, 45–50 mg/kg body weight) vs. vehicle for 5 consecutive days. To validate differential susceptibility after long-standing diabetes, urine albumin-to-creatinine ratio was measured 16 weeks after STZ in a separate group of mice. Open and closed circles indicate data from vehicle- and STZ-injected (i.e. control and diabetic) mice, respectively. Red and black circles indicate data from DN-susceptible and resistant mice, respectively. *, p-value < 0.05 in the same mouse strain between control and diabetic groups. #, p-value < 0.05 in the same treatment between the two mouse strains. Ctrl, control; DM, diabetic; N = 3–5 mice per group; n = 2 replicated wells per urine ELISA sample. (TIF) [file pone.0185250.s005.tif]

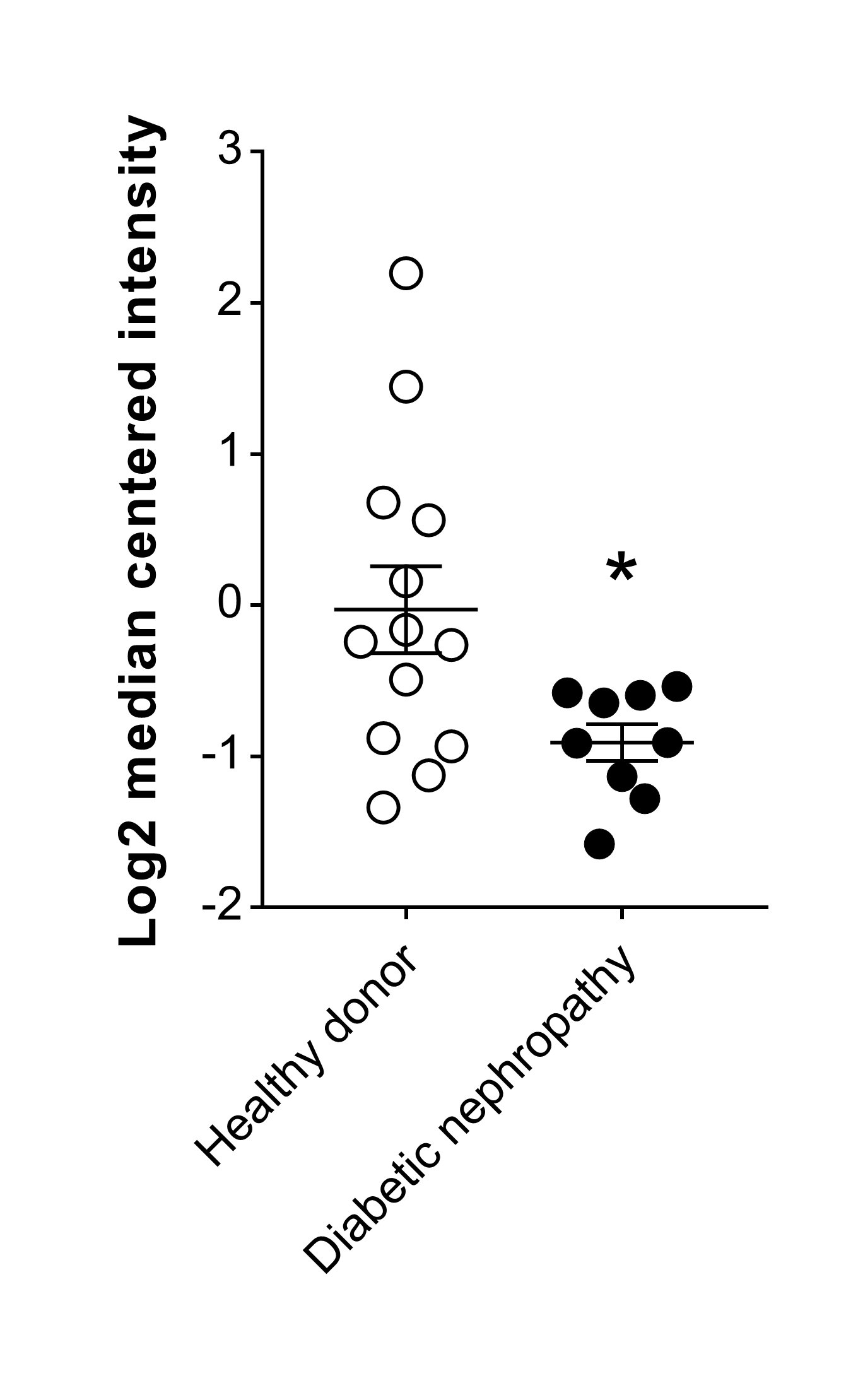

Supplement: S6 Fig — RNAseq data of Esm-1 is compared from the Nephroseq online resource. Open and closed circles indicate data from healthy human donors and individuals with DN, respectively. * p-value < 0.05. N = 9–13 individuals per group. (TIF) [file pone.0185250.s006.tif]
