# Supplementary material for: Murine glomerular transcriptome links endothelial cell-specific molecule-1 deficiency with susceptibility to diabetic nephropathy
Source: PLoS One. 2017 Sep 21;12(9):e0185250. doi: 10.1371/journal.pone.0185250 (PMC5608371; doi:10.1371/journal.pone.0185250)
Supplement: S1 Methods — (DOCX) [file pone.0185250.s007.docx]

**Supplementary Methods.**

**Transfection of HEK293T cells.** We plated 2 * 10^5^ HEK-293T cells in DMEM supplemented with 10% FCS without antibiotics. The next day, we transfected 3µg pCMV-mEsm-1-kan/Neo (Origene, Rockville, MD) or pT3-hEsm-1-V5 (kindly provided by Dr. Justin Annes, Endocrinology, Stanford) using Fugene HD (Promega, Madison, WI) in 6 well plates following the manufacturer's instructions. After 48 hours, we collected, aliquoted, and stored conditioned media.

**Expression data in Nephroseq.** We evaluated human glomerular Esm-1 mRNA in the publicly available Nephroseq database (https://www.nephroseq.org) from healthy donors and individuals classified with DN. We used Student’s t-test to compare these two groups. We expressed the results as mean +/- standard error of the mean and deemed differences to be statistically significant if the p-value was below 0.05.
