# Supplementary material for: Murine glomerular transcriptome links endothelial cell-specific molecule-1 deficiency with susceptibility to diabetic nephropathy
Source: PLoS One. 2017 Sep 21;12(9):e0185250. doi: 10.1371/journal.pone.0185250 (PMC5608371; doi:10.1371/journal.pone.0185250)
Supplement: S1 Table — (DOCX) [file pone.0185250.s008.docx]

**S1 Table.** Significantly Differentially Expressed genes in control vs. diabetic DN-susceptible mice.

| **Up-regulated Genes** | | **Down-regulated Genes** | |
| --- | --- | --- | --- |
| **Gene Name** | **Fold Change** | **Gene Name** | **Fold Change** |
| Ifit3 | 8.37 | Fmo5 | 0.50 |
| Ifi27l2a | 7.84 | C1qtnf3 | 0.50 |
| Cyp4a14 | 7.11 | Hsd17b11 | 0.47 |
| Lgals3bp | 6.89 | Akr1c14 | 0.46 |
| Irf7 | 5.15 | Slco1a1 | 0.43 |
| Kynu | 4.72 | Akr1c18 | 0.43 |
| Irgm2 | 4.27 | Chst7 | 0.42 |
| Gsta2 | 4.09 | Cyp4b1 | 0.41 |
| Igtp | 4.04 | Ces1f | 0.41 |
| Oas1g | 4.00 | Rbp1 | 0.39 |
| Cd74 | 3.99 | Tmem86a | 0.39 |
| H2-DMb1 | 3.82 | Slco1a4 | 0.37 |
| Irgm1 | 3.70 | Apoh | 0.37 |
| Ccnd1 | 3.55 | Bcat1 | 0.35 |
| Ifi27l1 | 3.30 | Acy3 | 0.35 |
| Ifi47 | 3.14 | Nudt19 | 0.35 |
| Gdf15 | 2.93 | Serpinf2 | 0.34 |
| Cdkn1a | 2.90 | Slc17a3 | 0.33 |
| Gldc | 2.83 | Cyp2j13 | 0.32 |
| Gbp3 | 2.83 | Mfsd2a | 0.30 |
| H2-Eb1 | 2.72 | Cyp7b1 | 0.27 |
| H2-Ab1 | 2.72 | Adh1 | 0.22 |
| Slc7a12 | 2.71 | Acsm3 | 0.17 |
| Cyp4a31 | 2.61 | Ccl28 | 0.17 |
| Spp1 | 2.57 | Hsd11b1 | 0.16 |
| Nox4 | 2.52 | Inmt | 0.14 |
| H2-DMa | 2.52 |  |  |
| Apol9b | 2.48 |  |  |
| Gbp2 | 2.37 |  |  |
| Gas6 | 2.34 |  |  |
| Mt1 | 2.34 |  |  |
| C3 | 2.33 |  |  |
| Oasl1 | 2.32 |  |  |
| B2m | 2.28 |  |  |
| Nuak2 | 2.26 |  |  |
| Acta1 | 2.22 |  |  |
| Irf9 | 2.21 |  |  |
| Prlr | 2.21 |  |  |
| Per2 | 2.18 |  |  |
| Rcan1 | 2.11 |  |  |
| Ccng1 | 2.07 |  |  |
| Nampt | 2.06 |  |  |
| Pigr | 2.04 |  |  |
| Tmem43 | 2.03 |  |  |
| Usp18 | 2.02 |  |  |
| Aldh1a1 | 2.01 |  |  |
| Smpdl3b | 1.99 |  |  |
