# Supplementary material for: Murine glomerular transcriptome links endothelial cell-specific molecule-1 deficiency with susceptibility to diabetic nephropathy
Source: PLoS One. 2017 Sep 21;12(9):e0185250. doi: 10.1371/journal.pone.0185250 (PMC5608371; doi:10.1371/journal.pone.0185250)
Supplement: S2 Table — (DOCX) [file pone.0185250.s009.docx]

**S2 Table.** Pathway analysis of Up- and Down-regulated pathways in control vs. diabetic DN-susceptible mice.

| **Network** | **P-Value** | **Min FDR*** |
| --- | --- | --- |
| Immune response_IFN-alpha/beta signaling via JAK/STAT | 1.518E-12 | 3.066E-10 |
| Immune response_IFN-alpha/beta signaling via MAPKs | 2.047E-07 | 1.749E-05 |
| Immune response_Lectin induced complement pathway | 3.035E-07 | 1.749E-05 |
| Immune response_Classical complement pathway | 4.329E-07 | 1.749E-05 |
| Immune response_Alternative complement pathway | 4.329E-07 | 1.749E-05 |
| Alternative complement cascade disruption in age-related macular degeneration | 2.459E-05 | 8.280E-04 |
| Immune response_Antiviral actions of Interferons | 1.940E-04 | 5.597E-03 |
| Immune response_Antigen presentation by MHC class II | 4.915E-04 | 1.241E-02 |
| Cell cycle_Regulation of G1/S transition (part 1) | 1.214E-03 | 2.647E-02 |
| Complement pathway disruption in thrombotic microangiopathy | 1.310E-03 | 2.647E-02 |
| Development_Role of Thyroid hormone in regulation of oligodendrocyte differentiation | 2.394E-03 | 4.182E-02 |
| Cell cycle_Nucleocytoplasmic transport of CDK/Cyclins | 2.675E-03 | 4.182E-02 |
| Rheumatoid arthritis (general schema) | 2.691E-03 | 4.182E-02 |
| Development_Prolactin receptor signaling | 4.105E-03 | 5.924E-02 |
| Development_Thrombopoetin signaling via JAK-STAT pathway | 6.599E-03 | 8.886E-02 |

^*^, Min FDR, Minimum false discovery rate.
