# Supplementary material for: Murine glomerular transcriptome links endothelial cell-specific molecule-1 deficiency with susceptibility to diabetic nephropathy
Source: PLoS One. 2017 Sep 21;12(9):e0185250. doi: 10.1371/journal.pone.0185250 (PMC5608371; doi:10.1371/journal.pone.0185250)
Supplement: S3 Table — (DOCX) [file pone.0185250.s010.docx]

**S3 Table.** Significantly Differentially Expressed genes in control vs. diabetic DN-resistant mice.

| **Up-regulated Genes** | | **Down-regulated Genes** | |
| --- | --- | --- | --- |
| **Gene Name** | **Fold Change** | **Gene Name** | **Fold Change** |
| Ifit3 | 4.18 | Fmo5 | 0.50 |
| Spink8 | 2.45 | Keg1 | 0.47 |
| Cdkn1a | 2.33 | C1qtnf3 | 0.46 |
| Aldh1a1 | 2.22 | Slco1a1 | 0.45 |
| Ifi27l1 | 2.16 | Odc1 | 0.39 |
| Phlda3 | 2.05 | Acsm2 | 0.39 |
| Aldh1a7 | 2.00 | Slco1a4 | 0.38 |
|  |  | BC026439 | 0.37 |
|  |  | Car3 | 0.37 |
|  |  | Gcnt1 | 0.36 |
|  |  | Ldhd | 0.35 |
|  |  | Cndp2 | 0.32 |
|  |  | Cyp4a12a | 0.30 |
|  |  | Serpinf2 | 0.30 |
|  |  | Nudt19 | 0.30 |
|  |  | Cyp2e1 | 0.29 |
|  |  | Slc17a3 | 0.29 |
|  |  | Hsd11b1 | 0.28 |
|  |  | Cyp2j13 | 0.28 |
|  |  | Azgp1 | 0.28 |
|  |  | Adh1 | 0.28 |
|  |  | Ces1f | 0.27 |
|  |  | Acy3 | 0.25 |
|  |  | Cyp4b1 | 0.24 |
|  |  | Ttr | 0.23 |
|  |  | Acsm3 | 0.17 |
|  |  | Inmt | 0.16 |
