# Supplementary material for: Murine glomerular transcriptome links endothelial cell-specific molecule-1 deficiency with susceptibility to diabetic nephropathy
Source: PLoS One. 2017 Sep 21;12(9):e0185250. doi: 10.1371/journal.pone.0185250 (PMC5608371; doi:10.1371/journal.pone.0185250)
Supplement: S4 Table — (DOCX) [file pone.0185250.s011.docx]

**S4 Table.** Pathway analysis of Up- and Down-regulated pathways in control vs. diabetic DN-resistant mice.

| **Network** | **P-Value** | **Min FDR*** |
| --- | --- | --- |
| Development_Retinoic acid and retinoic acid receptors in regulation of oligodendrocyte differentiation | 3.21E-05 | 2.44E-03 |
| Immune response_IL-4-induced regulators of cell growth, survival, differentiation and metabolism | 1.60E-04 | 4.24E-03 |
| Immune response_IFN-alpha/beta signaling via JAK/STAT | 1.67E-04 | 4.24E-03 |
| Retinol metabolism | 2.28E-04 | 4.33E-03 |
| Development_Role of Thyroid hormone in regulation of oligodendrocyte differentiation | 3.03E-03 | 4.60E-02 |
| Immune response_IFN-alpha/beta signaling via MAPKs | 7.62E-03 | 8.69E-02 |
| L-Arginine metabolism | 8.01E-03 | 8.69E-02 |
| DNA damage_Inhibition of telomerase activity and cellular senescence | 3.40E-02 | 1.25E-01 |
| Multiple sclerosis (general schema) | 3.57E-02 | 1.25E-01 |
| Development_Thrombopoetin signaling via JAK-STAT pathway | 3.74E-02 | 1.25E-01 |
| Immune response_IL-12 signaling pathway | 3.90E-02 | 1.25E-01 |
| Transcription_Transcription regulation of aminoacid metabolism | 4.24E-02 | 1.25E-01 |
| DNA damage_ATM / ATR regulation of G2 / M checkpoint | 4.40E-02 | 1.25E-01 |
| Ascorbate metabolism | 4.73E-02 | 1.25E-01 |
| Mitogenic action of Estradiol / ESR1 (nuclear) in breast cancer | 4.73E-02 | 1.25E-01 |

^*^, Min FDR, Minimum false discovery rate.
