# Supplementary material for: Murine glomerular transcriptome links endothelial cell-specific molecule-1 deficiency with susceptibility to diabetic nephropathy
Source: PLoS One. 2017 Sep 21;12(9):e0185250. doi: 10.1371/journal.pone.0185250 (PMC5608371; doi:10.1371/journal.pone.0185250)
Supplement: S5 Table — (DOCX) [file pone.0185250.s012.docx]

**S5 Table.** Significantly Differentially Expressed genes in control DN-susceptible vs. DN-resistant mice.

| **Up-regulated Genes** | | **Down-regulated Genes** | |
| --- | --- | --- | --- |
| **Gene Name** | **Fold Change** | **Gene Name** | **Fold Change** |
| Hdc | 12.77 | Arl5a | 0.50 |
| Tm4sf5 | 12.37 | Prkag2 | 0.49 |
| Angptl7 | 8.17 | Enpp5 | 0.49 |
| Ccl28 | 7.21 | Nudt6 | 0.48 |
| H2-Ea-ps | 4.65 | Manba | 0.48 |
| Lypd1 | 4.54 | 4833420G17Rik | 0.48 |
| 2810405K02Rik | 4.25 | Tmem70 | 0.48 |
| Fbxo2 | 4.10 | Stard7 | 0.47 |
| Mgst3 | 3.52 | Ctsc | 0.47 |
| Cap1 | 3.38 | Zfp68 | 0.47 |
| Rnase4 | 3.36 | Abcc2 | 0.47 |
| 6330403K07Rik | 3.32 | Zfand1 | 0.47 |
| Tpmt | 3.07 | Pdia3 | 0.46 |
| Gfer | 3.03 | Car3 | 0.46 |
| Ndn | 2.99 | Klk1 | 0.46 |
| Rbp1 | 2.98 | Copb1 | 0.45 |
| Acy1 | 2.96 | Fgfr1op2 | 0.45 |
| Hsd11b1 | 2.91 | Gmfb | 0.44 |
| Hpd | 2.90 | Slc22a4 | 0.44 |
| Crym | 2.89 | Tor1aip2 | 0.44 |
| Sult1c2 | 2.87 | Rbbp9 | 0.43 |
| Mfsd2a | 2.73 | Retsat | 0.42 |
| Rarres2 | 2.71 | Cdc42ep1 | 0.41 |
| Mpv17l | 2.66 | Tmem45b | 0.40 |
| Ang | 2.61 | Tmem66 | 0.40 |
| Pigz | 2.51 | Azgp1 | 0.38 |
| Tmem87a | 2.48 | Car4 | 0.38 |
| Serpina3b | 2.44 | Acox1 | 0.38 |
| Spink6 | 2.40 | Casp9 | 0.38 |
| Alad | 2.39 | H2-Ab1 | 0.37 |
| Cd82 | 2.35 | Rusc2 | 0.37 |
| Dbndd2 | 2.26 | Map3k4 | 0.36 |
| Rnase1 | 2.24 | Sspn | 0.36 |
| Aldh1a1 | 2.21 | Picalm | 0.34 |
| Rbm45 | 2.17 | Abcd3 | 0.34 |
| Hdhd3 | 2.16 | Sord | 0.32 |
| Stmn1 | 2.16 | Enpp2 | 0.32 |
| Glo1 | 2.11 | Thumpd1 | 0.32 |
| Acsm1 | 2.10 | Agtrap | 0.32 |
| Bcat2 | 2.09 | H2-Aa | 0.32 |
| Bcat1 | 2.09 | Ddah1 | 0.30 |
| Pfn1 | 2.09 | Mcm6 | 0.30 |
| Apoh | 2.07 | Snx5 | 0.29 |
| Hist1h4f | 2.04 | Klk1b4 | 0.28 |
| Anxa4 | 2.03 | Col1a2 | 0.27 |
|  |  | Klk1b8 | 0.26 |
|  |  | Psmb6 | 0.26 |
|  |  | Lpl | 0.25 |
|  |  | Klk1b27 | 0.24 |
|  |  | Cyp2d12 | 0.24 |
|  |  | Klk1b5 | 0.21 |
|  |  | Zfp330 | 0.20 |
|  |  | Aqp11 | 0.20 |
|  |  | Gsta2 | 0.19 |
|  |  | Rpl29 | 0.18 |
|  |  | Abhd16a | 0.13 |
|  |  | Me1 | 0.12 |
|  |  | Gcnt1 | 0.08 |
|  |  | Ttr | 0.05 |
