# Supplementary material for: Murine glomerular transcriptome links endothelial cell-specific molecule-1 deficiency with susceptibility to diabetic nephropathy
Source: PLoS One. 2017 Sep 21;12(9):e0185250. doi: 10.1371/journal.pone.0185250 (PMC5608371; doi:10.1371/journal.pone.0185250)
Supplement: S6 Table — (DOCX) [file pone.0185250.s013.docx]

**S6 Table.** Pathway analysis of Up- and Down-regulated pathways in control DN-susceptible vs. DN-resistant mice.

| **Network** | **P-Value** | **Min FDR*** |
| --- | --- | --- |
| Protein folding and maturation_Angiotensin system maturation \ Human version | 9.45E-11 | 2.15E-08 |
| Protein folding and maturation_Angiotensin system maturation \ Rodent version | 2.41E-10 | 2.74E-08 |
| Transport_Macropinocytosis regulation by growth factors | 5.73E-03 | 3.67E-01 |
| Immune response_IL-12 signaling pathway | 7.72E-03 | 3.67E-01 |
| Apoptosis and survival_p53-dependent apoptosis | 1.21E-02 | 3.67E-01 |
| Histamine metabolism | 1.21E-02 | 3.67E-01 |
| Apoptosis and survival_Granzyme B signaling | 1.55E-02 | 3.67E-01 |
| Nitrogen metabolism | 1.83E-02 | 3.67E-01 |
| Development_BMP7 in brown adipocyte differentiation | 2.13E-02 | 3.67E-01 |
| Immune response_Differentiation and clonal expansion of CD8+ T cells | 2.13E-02 | 3.67E-01 |
| Transcription_Sin3 and NuRD in transcription regulation | 2.34E-02 | 3.67E-01 |
| Apoptosis and survival_Apoptotic TNF-family pathways | 2.45E-02 | 3.67E-01 |
| Apoptosis and survival_Lymphotoxin-beta receptor signaling | 2.45E-02 | 3.67E-01 |
| Leucine, isoleucine and valine metabolism | 2.56E-02 | 3.67E-01 |
| Immune response_Antigen presentation by MHC class II | 3.08E-02 | 3.67E-01 |

^*^, Min FDR, Minimum false discovery rate.
