# Supplementary material for: Murine glomerular transcriptome links endothelial cell-specific molecule-1 deficiency with susceptibility to diabetic nephropathy
Source: PLoS One. 2017 Sep 21;12(9):e0185250. doi: 10.1371/journal.pone.0185250 (PMC5608371; doi:10.1371/journal.pone.0185250)
Supplement: S7 Table — (DOCX) [file pone.0185250.s014.docx]

**S7 Table.** Significantly Differentially Expressed genes in diabetic DN-susceptible vs. DN-resistant mice.

| **Up-regulated Genes** | | **Down-regulated Genes** | |
| --- | --- | --- | --- |
| **Gene Name** | **Fold Change** | **Gene Name** | **Fold Change** |
| Tm4sf5 | 8.97 | 1300014I06Rik | 0.50 |
| Mdk | 5.64 | Ctsc | 0.49 |
| Hdc | 4.48 | Map3k4 | 0.48 |
| Cyp4a12a | 4.22 | Casp9 | 0.47 |
| Hpd | 3.75 | Klk1b5 | 0.47 |
| Angptl7 | 3.71 | Zfp68 | 0.46 |
| Cyp4a12b | 3.28 | Agtrap | 0.45 |
| 2810405K02Rik | 3.02 | Csrnp1 | 0.44 |
| Sult1c2 | 2.99 | Thumpd1 | 0.42 |
| Sectm1b | 2.90 | Tsc22d3 | 0.41 |
| Hsd3b2 | 2.89 | Hmgn3 | 0.41 |
| Acy1 | 2.87 | Rasl11b | 0.38 |
| Tpmt | 2.68 | Mcm6 | 0.36 |
| Slc7a7 | 2.60 | Cd300lg | 0.36 |
| Tmem82 | 2.41 | Rusc2 | 0.33 |
| Crym | 2.37 | Snx5 | 0.32 |
| Lyplal1 | 2.35 | Me1 | 0.31 |
| Fam20b | 2.32 | Esm1 | 0.31 |
| Irgm1 | 2.32 | Cdc42ep1 | 0.30 |
| Adi1 | 2.29 | Sspn | 0.29 |
| Plau | 2.26 | Azgp1 | 0.27 |
| C3 | 2.26 | Ttr | 0.25 |
| Rnase4 | 2.23 | Zfp330 | 0.23 |
| Dpp7 | 2.22 | Psmb6 | 0.22 |
| Oas1g | 2.19 | Rpl29 | 0.19 |
| Prcp | 2.19 | Gcnt1 | 0.16 |
| Kynu | 2.19 | Abhd16a | 0.15 |
| Gfer | 2.17 |  |  |
| Ugt1a6a | 2.13 |  |  |
| Bcat2 | 2.12 |  |  |
| Ccbl2 | 2.08 |  |  |
| Apol9b | 2.06 |  |  |
| Gabrb3 | 2.05 |  |  |
| Pde1a | 2.01 |  |  |
