# Supplementary material for: Murine glomerular transcriptome links endothelial cell-specific molecule-1 deficiency with susceptibility to diabetic nephropathy
Source: PLoS One. 2017 Sep 21;12(9):e0185250. doi: 10.1371/journal.pone.0185250 (PMC5608371; doi:10.1371/journal.pone.0185250)
Supplement: S8 Table — (DOCX) [file pone.0185250.s015.docx]

**S8 Table.** Pathway analysis of Up- and Down-regulated pathways in diabetic DN-susceptible vs. DN-resistant mice.

| **Network** | **P-Value** | **Min FDR*** |
| --- | --- | --- |
| Immune response_Lectin induced complement pathway | 1.53E-08 | 8.68E-07 |
| Immune response_Classical complement pathway | 2.19E-08 | 8.68E-07 |
| Immune response_Alternative complement pathway | 2.19E-08 | 8.68E-07 |
| Alternative complement cascade disruption in age-related macular degeneration | 3.50E-06 | 1.04E-04 |
| Complement pathway disruption in thrombotic microangiopathy | 3.17E-04 | 7.53E-03 |
| L-Methionine metabolism | 1.07E-03 | 2.13E-02 |
| Immune response_IL-4-induced regulators of cell growth, survival, differentiation and metabolism | 1.30E-03 | 2.21E-02 |
| Apoptosis and survival_p53-dependent apoptosis | 4.44E-03 | 6.60E-02 |
| Cortisol biosynthesis from Cholesterol | 7.54E-03 | 9.97E-02 |
| K-RAS signaling in pancreatic cancer | 1.00E-02 | 1.19E-01 |
| Cortisone biosynthesis and metabolism | 1.33E-02 | 1.37E-01 |
| Immune response_Antiviral actions of Interferons | 1.38E-02 | 1.37E-01 |
| Androstenedione and testosterone biosynthesis and metabolism p.1 | 1.59E-02 | 1.45E-01 |
| Immune response_IFN-alpha/beta signaling via MAPKs | 2.89E-02 | 2.33E-01 |
| Androgen biosynthetic pathways | 3.24E-02 | 2.33E-01 |

^*^, Min FDR, Minimum false discovery rate.
