# Supplementary material for: Murine glomerular transcriptome links endothelial cell-specific molecule-1 deficiency with susceptibility to diabetic nephropathy
Source: PLoS One. 2017 Sep 21;12(9):e0185250. doi: 10.1371/journal.pone.0185250 (PMC5608371; doi:10.1371/journal.pone.0185250)
Supplement: S9 Table — (DOCX) [file pone.0185250.s016.docx]

**S9 Table.** Comparing expression by Microarray and qPCR.

| **Transcript** | **Genbank No.** | **Microarray** | | **qPCR** | |
| --- | --- | --- | --- | --- | --- |
|  |  | **Amplification site** | **Fold change** | **Amplification site** | **Fold change** |
| Esm-1 | NM_023612.3 | 1987-2036 | 0.31* | 1218-1397 | 0.09* |
| Tsc22d3 | NM_001077364.1 | 1972-2021 | 0.41* | 1246-1396 | 0.51 |
| Midkine | NM_010784.5 | 964-1013 | 5.64* | 603-767 | 5.72* |

*, P-Value < 0.05
